# Mogroside V Alleviates Concanavalin A-induced Acute Liver Injury by Inhibiting Inflammatory Responses and M1 Macrophage Polarization

**DOI:** 10.1007/s10753-026-02493-8

**Published:** 2026-03-29

**Authors:** Yuxuan Zhao, Zhihong Liu, Fenglian Yan, Hongru Zhao, Xinzhou Xie, Jiaying Li, Hui Zhang, Lin Wang, Jia Fu, Chunxia Li, Jun Dai, Huabao Xiong, Bin Yu, Junfeng Zhang

**Affiliations:** 1 Institute of Immunology and Molecular Medicine, Jining Medical University, Jining, 272067 China; 2 School of Basic Medicine, Shandong First Medical University, Jinan, 271016 China; 3 Jining Key Laboratory of Immunology, Jining Medical University, Jining, 272067 China; 4 Department of Spine Surgery, Jining First People’s Hospital, Jining, 272011 China; 5 Basic Medical College, Jining Medical University, Jining, 272067 China; 6 College of Integrated Chinese and Western Medicine, Jining Medical University, Jining, 272067 China

**Keywords:** Mogroside V, Concanavalin A, Acute liver injury, M1 macrophage

## Abstract

Acute liver injury is a highly lethal condition characterized by hepatocyte damage and dysregulation of immune homeostasis. In this study, we aimed to identify the pharmacological effects and mechanisms of action of Mogroside V (Mog V), an active triterpenoid saponin derived from *Siraitia grosvenorii* exhibits protective effects in acute liver injury. We used concanavalin A (Con A) to induce acute liver injury in C57BL/6J mice and established both preventive and therapeutic intervention models. The experimental results showed that: In the preventive model, mice were intraperitoneally injected with Mog V 3 h prior to Con A (20 mg/kg, tail vein injection); in the therapeutic model, Mog V was administered 1 h after Con A challenge. In both models, Mog V treatment led to a marked reduction in serum aminotransferase (ALT/AST) levels, as well as attenuated hepatocellular necrosis and inflammatory cell infiltration. Network pharmacology analysis identified several relevant biological pathways associated with inflammation and oxidative stress. Mog V reduced oxidative damage by restoring peroxide levels and reducing liver cell apoptosis and modulated immune responses by suppressing the activation of proinflammatory M1 macrophages and reducing cytokine levels. The results of a macrophage adoptive transfer assay indicated that Mog V-treated macrophages alleviated liver injury and inflammation in mice and suppressed the activity of key inflammation-related signaling pathways. These findings suggest that Mog V has the potential to be a promising candidate for the development of therapeutic agents against Con A-induced acute liver injury, and further studies are needed to verify its clinical application potential.

**Supplementary Information:**

The online version contains supplementary material available at 10.1007/s10753-026-02493-8.

## Background

Acute liver injury has a variety of triggers, including adverse drug reactions, excessive alcohol intake, sepsis, and infections caused by hepatitis viruses or bacteria, with immune activation being a common feature. This immune response includes immune cell activity, inflammatory cytokine release, and marked increases in aspartate transaminase (AST) and alanine transaminase (ALT) levels [1]. In murine models, intravenous administration of concanavalin A (Con A) is effective in inducing acute liver injury. The hallmark features of this disorder include the persistent recruitment of macrophages and CD4^+^T cells into the liver and the occurrence of infiltration [2, 3]. Liver macrophages undergo a process of differentiation into M1 macrophages, which are capable of releasing a range of cytokines, such as tumor necrosis factor alpha (TNF-α), interleukin 6 (IL-6), and IL-12, that promote inflammatory responses and induce severe liver inflammation, potentially irreversible liver damage and dysfunction [4]. Given that the mouse model of Con A-induced acute liver injury is highly similar to the pathological features of human liver injury, this model has become a standardized tool for exploring the underlying mechanisms of acute liver injury [5].

The liver is the most important metabolic and detoxification organ in the body and contains many types of immune cells (e.g., macrophages, Kupffer cells, and lymphocytes), which are essential for pathogen removal and immune regulation [6]. The involvement of immune cells and the discharge of inflammatory factors largely determine the degree of liver damage [7]. Hepatic macrophages, including Kupffer cells and monocyte-derived and fetal-origin resident macrophages, comprise 20%–35% of the liver’s non-parenchymal cells [8, 9]. Given the pivotal roles of these macrophages in both the liver and systemic intrinsic and adaptive immunity, the involvement of macrophage activation in immune-mediated acute liver injury appears to be of particular importance [10, 11]. In response to lipopolysaccharide (LPS) and interferon gamma (IFN-γ) stimulation, macrophages differentiate into classical M1 macrophages and discharge a variety of inflammation-related cytokines, including IL-1β, IL-6, and TNF-α [12], or into M2 macrophages, which are activated by IL-13/IL-4 to release anti-inflammatory cytokines (e.g., IL-10 and TGF-β) [13]. Macrophage-generated superoxide significantly enhances cytokine release levels in Con A-induced liver injury [14]. TNF-α and IFN-γ (interferon-γ) have been determined to be critical mediators of Con A-induced acute liver injury [15, 16], with macrophages being the primary source. Therefore, the severity of Con A-induced liver injury may be effectively attenuated by modulating macrophage activation.

*Siraitia grosvenorii* is a common therapeutic and dietary plant native to southern China [17]. *S. grosvenorii* contains Mogroside V (Mog V), a triterpenoid saponin with antioxidant, anti-inflammatory, and antitumor properties [18, 19]. Mog V modulates oxidative stress by mediating glutathione (GSH), purine, and riboflavin metabolism [19]; The latter plays a vital role in macrophage activation and immune response modulation [20]. Mog V can also ameliorate myeloperoxidase (MPO) levels and alleviate histological injury in acute lung injury by suppressing inducible nitric oxide synthase (iNOS) and cyclooxygenase-2 (COX-2), reducing Inhibitor kappa B-alpha (IkBa) phosphorylation, and blocking nuclear factor kappa-B (NF-κB) [21]. Mog V also reduces the levels of signal transducer and activator of transcription 3 (STAT-3) and extracellular regulated protein kinases (ERK) phosphorylation, thereby reducing tumor growth [22, 23]. Thus, Mog V has been reported to exert regulatory effects on immune and inflammatory responses in several disease models, such as allergic pneumonitis and cancer. However, the potential protective effects and underlying molecular mechanisms of Mog V in Con A-induced acute liver injury have not been previously investigated. Consequently, in this study, we examined the mechanisms by which macrophages intensify acute liver injury and investigated the pharmacological actions and mechanisms of Mog V using a Con A-induced liver injury model.

## Methods

### Animals and Reagents

For the experimental animals, we used 6- to 8-week-old male C57 BL/6J mice (weighing approximately 20 g) obtained from Jinan Pengyue Experimental Animal Breeding Company (Jinan, China) and maintained for 1 week in a temperature-controlled pathogen-free environment prior to conducting experiments. All animal experiments were conducted in compliance with the Code of Laboratory Animal Husbandry and Handling, which has been endorsed by the Ethical Review Commission for Laboratory Animals of Jining Medical College.

Mog V (Product No.: HY-N0502, purity: 99.01%) was purchased from MedChemExpress, and Con A (Catalog No.: C2010) was purchased from Sigma-Aldrich (St. Louis, MO, USA). The Nanjing Jianjian Institute of Biotechnology and Engineering (Nanjing, China) provided the kits used for superoxide dismutase (SOD), malondialdehyde (MDA), glutathione (GSH), and MPO analyses. The enzyme-linked immunosorbent assay (ELISA)-related reagents for TNF-α, IL-12, and IL-6, as well as the antibodies for flow cytometric analysis, were provided by BioLegend (San Diego, CA, USA). All reagents used for qRT-PCR analyses were obtained from Vazyme (Nanjing, China). The specific reagents used in this study included HiScript III Reverse Transcription Supermix (Product Code: R323) and AceQ qPCR SYBR Green premix (Product Number: Q111). Radio immunoprecipitation assay (RIPA) lysis buffer (Part No. P0013 B), Boosted Bicinchoninic Carboxylic Acid (BCA) Protein Assay Kit (Part No. P0010), TdT-Mediated dUTP Notched End Labeling (TUNEL) Assay Kit (Part No. 1088), anti-β-actin (Product No. AA128), horseradish peroxidase (HRP)-labeled goat anti-mouse IgG (Product No. A0216), and HRP-labeled goat anti-rabbit IgG (Product No. A0208) antibodies were supplied by Beyotime (Shanghai, China). Cell Signaling, Inc. (Boston, MA, USA) provided JNK (Product No. 9252), pJNK (Product No. 4668), ERK (Product No. 4695), p-ERK (Product No. 4370), p38 (Product No. 8690), phosphorylated p38 (p-p38 (Product No.: 4511), p65 (Product No.: 8242), and phosphorylated p65 (p-p65; Product No.: 3033) antibodies.

### Model Construction and Drug Administration

In accordance with the experimental design, C57BL/6J mice were randomly assigned to one of the following eight treatment groups: control, 2.5 mg/kg Mog V (Mog-V-2.5-mg/kg), 5 mg/kg Mog V (Mog-V-5-mg/kg), 10 mg/kg Mog V (Mog-V-10-mg/kg), Con A (Con A), 2.5 mg/kg Mog V pre-treated Con A (2.5-mg/kg + Con A), 5 mg/kg Mog V pre-treated Con A (5-mg/kg + Con A), and 10 mg/kg Mog V pre-treated Con A (10-mg/kg + Con A). To evaluate the preventive effect of Mog V on Con A-induced acute liver injury: mice in the experimental groups were injected intraperitoneally with different concentrations of Mog V, whereas those in the control group were injected with an equal amount of phosphate-buffered saline (PBS). After 3 h, mice were administered a dose of Con A (20 mg/kg) via the tail vein. Twelve hours later, all mice were euthanized under narcosis, and serum and hepatic tissue samples were collected. To confirm the therapeutic effect of Mog V on Con A-induced acute liver injury, mice were first injected with Con A to induce acute liver injury. One hour after Con A challenge, Mog V was administered via intraperitoneal injection. Samples were collected 12 h after Con A injection for subsequent detection. For the mouse survival experiments, mice were assigned to the Con A group, 2.5 mg/kg Mog V + Con A group, 5 mg/kg Mog V + Con A group, and 10 mg/kg Mog V + Con A group. Intraperitoneal injections of PBS or different doses of Mog V were administered to each group of mice. After 3 h, a lethal concentration of Con A (25 mg/kg) was administered through the tail vein, and the time until death was recorded.

### Network Pharmacology Analysis

Screening for potential targets of Mog V using the PharmMapper database [24] yielded 299 candidate drug targets. By setting a normal fit value threshold of ≥ 0.5, we identified approximately 155 potential Mog V target genes and obtained the gene designations of these targets with reference to the UniProt database. We searched the following databases for potential target genes using the keyword “acute liver injury”: Online Mendelian Inheritance in Man (OMIM-Gene-Map, downloaded on 2024, 05, 10) [25], DisGeNET (DisGeNET version 7.0), DrugBank (version 5.1.10, update date: January 4, 2023), and GeneCards (GeneCards version 5.20 update date: May 14, 2024) [26]. By referring to the bioinformatics website (http://www.bioi-informatics.com.cn), we obtained a Wayne diagram of the relationship between Mog V targets and acute liver injury and the commonly associated genes. In addition, we constructed protein–protein interaction (PPI) network models using information provided by the STRING website (https://cn.string-db.org/) based on a confidence level of ≥ 0.4, and used Cytoscape for intuitive visualization of the PPI networks. Furthermore, we performed functional enrichment analysis of the Kyoto Encyclopedia of Genes and Genomes (KEGG) and Gene Ontology (GO) biological processes using the Metascape online platform (https://metascape.org/) for the commonly associated genes.

### ELISA to Determine Serum Cytokine Levels

ELISA kits (BioLegend) were used to determine the levels of TNF-α, IL-12, and IL-6 expression. The ELISA plates were initially pre-treated with diluted capture antibody and then washed with PBS containing 0.05% Tween 20 after overnight refrigeration at 4 °C. Subsequently, the standards and samples were appropriately diluted and added to the wells. After a 2-h incubation at 37 °C, 100 µL of detection antibody was added to each well. Following a further 1-h incubation and subsequent washing, an anti-biotin protein–HRP complex was added, and the mixture was incubated for 30 min. Substrate solution was then added and incubated for approximately 15–30 min before the reaction was terminated using the stop solution. The absorbance values of the wells were recorded at 450 nm using an enzyme-linked immunosorbent assay (ELISA) meter.

### Pathological Analysis of Tissue Sections

#### H&E Staining and Statistical Analysis of Necrotic Areas

After removing the mouse livers, 4% paraformaldehyde was immediately added for fixation, followed by paraffin embedding, sectioning, and hematoxylin-eosin (H&E) staining as previously described [27]. H&E-stained sections were observed under a light microscope (Olympus, Tokyo, Japan). The necrotic area ratio was quantified using ImageJ software, with the calculation formula: Ratio of necrotic area (%) = (Area of necrotic region / Total area of liver section field of view) × 100%.

#### Immunofluorescence Staining

Paraffin-embedded liver tissue sections were first dewaxed with xylene, followed by hydration through a gradient ethanol series. Antigen retrieval was then performed via microwave heating. To eliminate endogenous peroxidase activity, sections were treated with 3% hydrogen peroxide (H₂O₂) at room temperature for 20 min. Next, sections were incubated overnight at 4 °C with the primary antibody against CD86. After thorough washing with PBS, sections were incubated with the corresponding HRP-labeled secondary antibody at room temperature for 1 h. Finally, sections were mounted using DAPI-containing anti-fluorescence quenching mounting medium and imaged under a fluorescence microscope (Olympus Optical, Tokyo, Japan).

#### Immunohistochemical Staining

Paraffin sections were baked at 60 °C for 2 h, dewaxed with xylene, and hydrated through a gradient ethanol series. Antigen retrieval was performed in citrate buffer at high temperature for 15 min. Endogenous peroxidase was blocked with 3% H₂O₂ at room temperature for 10 min, followed by 3 washes with PBS. Sections were then blocked with 5% BSA at room temperature for 30 min; the blocking solution was discarded, and primary antibody working solution was added, with incubation overnight at 4 °C in a humidified chamber. The next day, sections were warmed to room temperature for 30 min and rinsed with PBS. HRP-labeled secondary antibody was added and incubated at room temperature for 30 min. After washing with PBS, sections were developed with DAB for 3–5 min, and the reaction was terminated promptly. Sections were counterstained with hematoxylin for 30 s, differentiated with hydrochloric acid-ethanol, blued, dehydrated through a gradient ethanol series, cleared with xylene, mounted with neutral resin, and observed/photographed under a microscope.

### Isolation of Single-nucleated Liver Cells

Single-nucleated liver cells were isolated according to a previously detailed procedure [28]. Initially, 20 mL of PBS was perfused into the left ventricle and allowed to flow through to the liver via a minor cut in the right atrium of the mouse. The livers were collected and ground, and the obtained hepatic homogenates were passed through a stainless-steel 200-mesh sieve. The filtered hepatic homogenate was centrifuged at 1000 × g for 10 min. Afterward, the precipitate was resuspended in PBS buffer and centrifuged at 20 × g for 5 min. The supernatants were centrifuged at 700 ×*g* for 8 min to yield pellets containing hepatocytes. The pellets were resuspended in 40% Percoll solution (3 mL) and gently layered onto 70% Percoll solution. Subsequently, after centrifugation for 30 min at 700 ×*g*, liver mononuclear cells were isolated from the middle layer and washed twice with PBS.

### Culture of Bone Marrow-derived and M1 Macrophages

Bone marrow cells were extracted from the leg bones of wild-type mice, filtered on an ultra-clean bench, and erythrocytes were removed using an erythrocyte lysis solution. To promote macrophage development, the remaining cells were resuspended in culture medium and treated with macrophage colony-stimulating element (GM-CSF, 10 ng/mL) for 7 days. On day 7, LPS (200 ng/mL) and IFN-γ (10 ng/mL) were added to the medium to stimulate the transition of M0- to M1-type macrophages.

### Determination of Biochemical Indicators

To quantify serum ALT and AST levels, mouse serum was diluted 10-fold in saline and analyzed using a Cobas 8000 multibiochemical analyzer (Roche, Basel, Switzerland). The levels of MPO, GSH, MDA, and SOD in mouse liver homogenates were measured using commercially available kits following the protocols provided by the manufacturers. Following induction of macrophage differentiation into M1 macrophages and subsequent treatment with Mog V, cellular proteins were collected using RIPA lysis buffer containing protease inhibitors. Total protein concentration was determined using a BCA protein quantification kit. Subsequently, commercially available kits were used to measure GSH, SOD, MDA, and MPO activity in macrophages to clarify the regulatory effect of Mog V on the oxidative stress response of M1 macrophages.

### Cell Viability Assay

The culture medium containing macrophages (5 × 10^4^ cells/well) was added to the wells of 96-well plates containing different concentrations of Mog V (50, 100, 200, 400, 800, and 1000 µM), and M1-type macrophages were induced by exposure to LPS (200 ng/mL) and IFN-γ (10 ng/mL) for 3 h. One day later, the viability of the induced M1-type macrophages was assessed using a Cell Titer Lumi Luminescent Cell Viability Assay Kit (Beyotime).

### Flow Cytometric Analysis

Bone marrow-derived macrophages and hepatic mononuclear cells (HMNCs) were collected in flow tubes containing the relevant antibodies. Cells were incubated in the dark at 4 °C for 30 min, washed with PBS, and analyzed by flow cytometry using a BD FACSVerse flow cytometric analyzer (Becton Dickinson, New Jersey, USA) to determine differences in cell proportions.

### Quantitative Real-time Polymerase Chain Reaction (PCR)

RNA was obtained from cultured cells or mouse livers using TRIzol reagent (TaKaRa, Japan) and then reverse-transcribed into cDNA using HiScript III RT Super Mix (Vazyme). qPCR was performed using an AceQ qPCR SYBR Green Master Mix (Vazyme) and a Light Cycler 480 system (Roche) according to the manufacturer’s instructions, with the following primer pairs used for amplification: TNF-α forward 5ʹ-GCCACCACGCTCTTCTGTCT-3ʹ and reverse 5ʹ-GGTCTGGGCCATAGAACTGATG-3ʹ; glyceraldehyde-3-phosphate dehydrogenase (GAPDH) forward 5′-AACGACCCCTTCATTGAC-3′ and reverse 5-TCCACGACATACTCAGCAC-3; IL-12 forward 5ʹ-AGACATGGGAGTCATAGGCTCTG- 3ʹ and reverse 5ʹ-CCATTTTCCTTCTTGTGTGAGCA-3ʹ; and IL-6 forward 5ʹ-CCAGAAACC GCTATGAAGTTCCT-3ʹ and reverse 5ʹ-CACCAGCATCA GTCCCAAGA-3ʹ.

### Macrophage Adoptive Transfer

Macrophages were cultured in vitro using a previously described procedure [28]. To verify which administration route (intraperitoneal injection or tail vein injection) is more conducive to the homing of macrophages to the liver, bone marrow-derived macrophages cultured in vitro were labeled with CFSE (1 µM, incubated for 20 min in the dark), and the cell density was then adjusted to 2 × 10⁷ cells/mL. C57BL/6 mice were divided into 5 groups: the Control group, the intraperitoneal macrophage injection group (i.p.+Mø, injected with CFSE⁺ macrophages), the tail vein macrophage injection group (i.v.+Mø, injected with CFSE⁺ macrophages), the intraperitoneal macrophage injection + Con A group (i.p.+Mø+Con A), and the tail vein macrophage injection + Con A group (i.v.+Mø+Con A). Mice in each group were injected with 2 × 10⁶ macrophages per mouse via intraperitoneal or tail vein injection, respectively. Twelve hours later, Con A was injected via the tail vein to induce liver injury. Mice were euthanized 12 h after Con A injection, and liver tissue samples were collected. Intraperitoneal injection demonstrated superior liver homing efficiency compared to tail vein injection, indicating that intraperitoneally injected macrophages effectively migrate to the liver (Supplementary Fig. 1A-D). To investigate whether macrophages treated with Mogroside V (Mog V) exert critical biological effects, obtained cells were pre-treated with Mog V (200 µM). Mice were randomly allocated to one of four treatment groups (Mϕ, Mog-V + Mϕ, Con-A + Mϕ, and Con-A + Mog-V + Mϕ) and subsequently intraperitoneally injected with macrophages (2 × 10^6^/mouse). Following a 12 h treatment, Con A (20 mg/kg) was administered via tail injection, and the mice were euthanized after a further 12 h, at which point serum samples and hepatic tissues were collected. Number of CFSE⁺ cells in liver = CFSE (%) × Total number of single-nucleated liver cells. Homing efficiency (%) = (Number of CFSE⁺ cells in liver / Total number of injected CFSE⁺ macrophages) × 100%.

### Western Blotting

The harvested macrophages were lysed using a mixture of RIPA buffer (Product No. P0013 B, Beyotime) and protease inhibitors (EDTA, PI, and PMSF). The protein concentrations were quantified using an enhanced BCA protein assay kit. Protein specimens were separated on 10% SDS-PAGE gels, which were subsequently transferred to a 0.45-µm PVDF film. The membranes were blocked in 5% bovine serum albumin for 2 h and incubated with the appropriate primary antibodies overnight at 4 °C. The next day, after washing three times with TBST (10 min/wash), the films were incubated with HRP-tagged secondary antibodies for 1.5 h, after which they were treated using an enhanced chemiluminescence kit (Biosharp, Hefei, China) and developed using chemiluminescence equipment (General Electric Company, CT, USA).

### Statistical Analysis

Data were analyzed using GraphPad Prism software (version 9.0) and are presented as mean values and standard error ranges (expressed as mean ± standard error). One-way analysis of variance (ANOVA), two-way ANOVA, or Student’s t-tests were used to assess differences between groups. For the mouse survival experiments, analyses were executed using the Kaplan–Meier method and chronological test. Statistically significant differences are expressed as follows: *p < 0.05, **p < 0.01, and ***p < 0.001.

## Results

### Mog V Attenuates Con A-induced Liver Injury in Mice

**Fig. 1:**
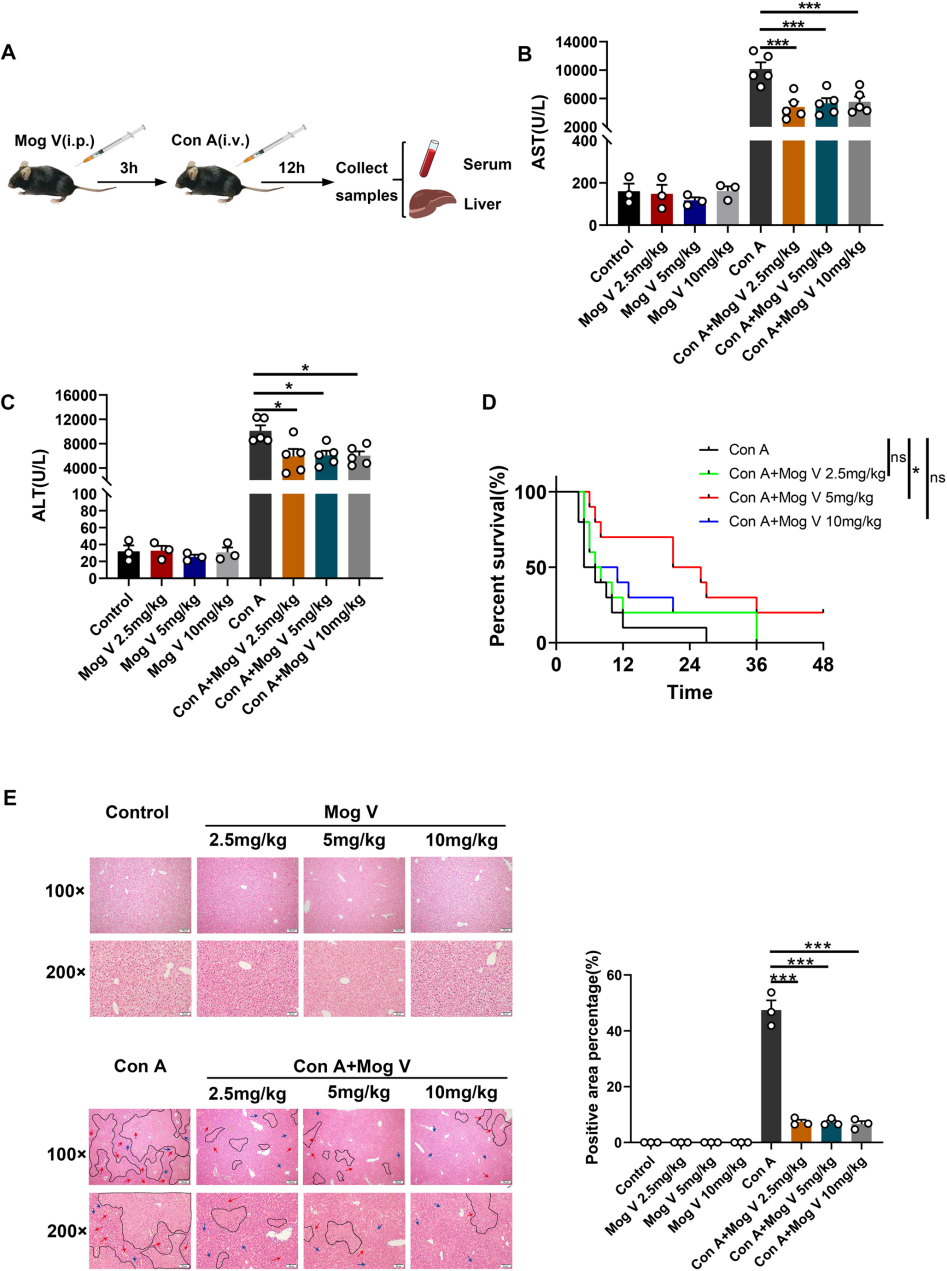
Mogroside V (Mog V) protects mice from concanavalin A (Con A)-induced acute liver injury. Mice were administered intraperitoneal injections of Mog V (2.5, 5, 10 mg/kg), followed by tail vein injections of Con A (20 mg/kg) 3 h later. After 12 h, serum and livers were collected. (**A**) Flow chart of drug administration. (**B**) Serum aspartate transaminase (AST) levels. (**C**) Serum alanine aminotransferase (ALT) levels. (**D**) Survival curve of mice treated with a lethal dose of Con A (25 mg/kg, independent of the 20 mg/kg model in A-C; *n* = 10). (**E**) Quantitative analysis was performed for the percentage of pathological lesion areas in H&E-stained liver sections (original magnification: ×100 and ×200) and the percentage of H&E-positive areas. Necrotic regions are marked with black dashed lines, areas of congestion with red arrows, and sites of inflammatory cell infiltration with blue arrows. Data are presented as the mean ± standard error of the mean (SEM). ns: Not significant, *p < 0.05, ***p < 0.001

To investigate the principle of action of Mog V and determine the dose-dependent action in acute liver injury, we used Con A to injure mouse livers and assessed the influence of Mog V concentration and duration of administration. A flowchart of the experiment is presented in Fig. 1A. Mice were intraperitoneally injected with Mog V at concentrations of 2.5, 5, and 10 mg/kg, followed by a tail vein injection of Con A (20 mg/kg). Mog V treatment markedly decreased the Con A-induced increase in serum aminotransferase AST and ALT levels and had no adverse effects on the liver (Fig. 1B, C). To further investigate the dose dependency of Mog V’s effects, mice were pretreated with Mog V prior to injection of a lethal dose of Con A (25 mg/kg). The results indicated that 5 mg/kg Mog V significantly improved mouse survival (Fig. 1D). Combined with histological and biochemical analyses confirming comparable protective efficacy among the three doses, 5 mg/kg was selected as the optimal dose for subsequent in vivo experiments to balance efficacy, safety, and experimental validity. To further determine the mechanisms by which Mog V modulates the inflammatory response in liver injury, H&E staining of liver sections was performed to examine the degree of hepatocyte degeneration and necrosis. Although liver tissues obtained from mice in the control and drug-alone groups appeared healthy, mice in the Con A-treated group showed significant hepatocellular necrosis in the region around the central vein of the liver, which was accompanied by the infiltration of a large number of inflammatory cells. The extent of necrotic hepatic tissue was markedly reduced in Mog V-treated mice (Fig. 1E). These findings indicate that pretreatment with Mog V attenuates the pathological changes in patients with acute liver injury attributable to Con A. To further explore whether Mog V exerts a therapeutic effect on established acute liver injury (beyond its preventive role), we conducted a supplementary experiment with a modified intervention timeline (a flowchart of the experiment is presented in Supplementary Fig. 2A). Results demonstrate that serum ALT and AST levels (Supplementary Fig. 2B), alongside H&E staining of liver tissue (Supplementary Fig. 2C), collectively indicate that Mog V retains its hepatoprotective effects when administered after the establishment of the injury model. This finding indicates that Mog V exerts protective effects in both preventive and therapeutic intervention models of Con A-induced liver injury in mice.

### Network Analysis of Mog V Pharmacokinetics

**Fig. 2:**
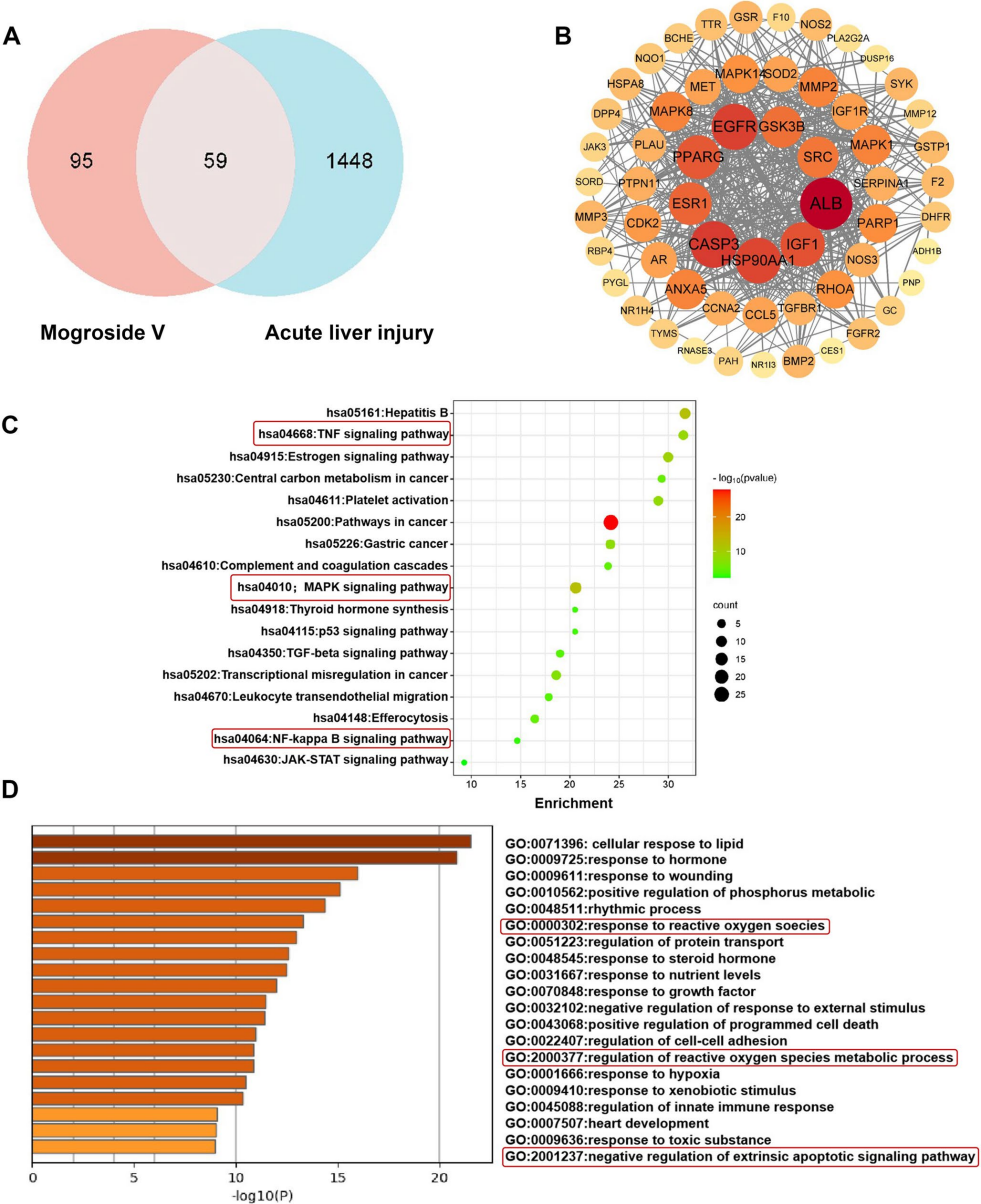
Network pharmacology analysis of Mog V. Potential targets of Mog V were screened using databases, and Venn diagrams as well as shared genes between Mog V targets and those associated with acute liver injury were obtained from bioinformatics websites. A protein-protein interaction (PPI) network was constructed and visualized using Cytoscape. Additionally, the Metascape platform was employed to perform Kyoto Encyclopedia of Genes and Genomes (KEGG) pathway enrichment analysis and Gene Ontology (GO) biological process enrichment analysis on the shared genes. (**A**) Common target genes of Mog V and acute liver injury. (**B**) Protein interaction network of common genes. (**C**) Kyoto Encyclopedia of Genes and Genomes (KEGG) analysis of common genes. (**D**) Gene Ontology analysis of the biological process enrichment of common genes

Based on our search of the Pharmapper database, we identified 299 potential targets of Mog V, of which 155 were retained after a screening value of ≥ 0.5. A total of 1507 disease targets associated with acute liver injury were obtained using four databases, and 59 of these intersected with the targets of Mog V (Fig. 2A). Construction of a PPI network based on these targets using Cytoscape software revealed the following nine key genes: epidermal growth factor receptor (*EGFR*), *GSK3B*, non-receptor tyrosine kinase (*SRC*), *IGF1*, caspase-3 (*CASP3*), peroxisome proliferator-activated receptor γ (*PPARG*), heat shock protein 90α (*HSP90AA1*), *ALB*, and estrogen receptor 1 (*ESR1*) (Fig. 2B). To verify the network pharmacology-predicted targets, we detected the mRNA expression of ALB, EGFR, and ESR1 in liver tissues via qPCR. Compared with the Control group, ALB mRNA was significantly downregulated in the Con A group, while Mog V pretreatment partially restored its expression (Supplementary Fig. 3A). For EGFR mRNA, Con A stimulation induced a dramatic upregulation, and this effect was reversed by Mog V (Supplementary Fig. 3B). In addition, ESR1 mRNA was notably reduced in the Con A group, and Mog V pretreatment alleviated this decrease (Supplementary Fig. 3C). These results suggest that Mog V can modulate the mRNA expression levels of the predicted key targets (ALB, EGFR, ESR1) in the liver tissues of mice with Con A-induced acute liver injury. KEGG analysis, using intersecting targets in Metascape, revealed NF-κB, TNF-α, and MAPK pathways to be key signaling pathways and that the identified genes grouped in 17 clusters that included numerous different pathways (Fig. 2C). The GO richness analysis suggested that the effect of Mog V on acute liver injury is related to the regulation of metabolic processing via oxidative stress, including the response to reactive oxygen species (ROS), regulation of ROS metabolic processes, and negative regulation of the extrinsic apoptotic signaling pathway (Fig. 2D), thereby providing evidence that Mog V attenuates acute Con A-mediated liver injury by modulating oxidative stress.

### Mog V Ameliorates Hepatic Cell Apoptosis and Oxidative Stress

**Fig. 3:**
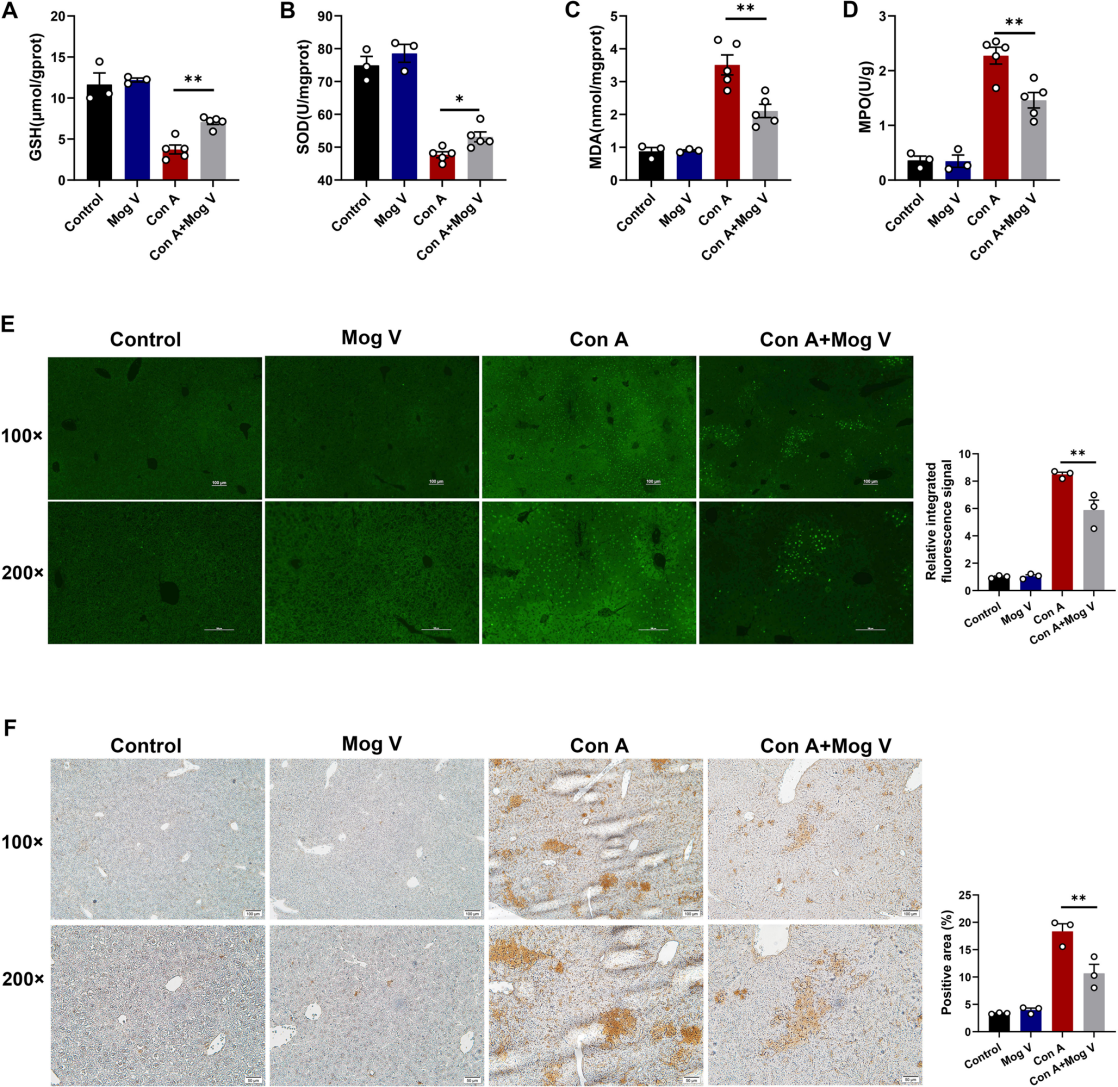
Mog V ameliorates Con A-induced oxidative stress and hepatocyte apoptosis in liver tissues. Mog V alleviates Con A-induced oxidative stress and hepatocyte apoptosis in liver tissue. Mice received intraperitoneal injections of Mog V (5 mg/kg), followed by Con A (20 mg/kg) challenge 3 h later. Liver tissues were collected 12 h later. Levels of (**A**) glutathione (GSH), (**B**) superoxide dismutase (SOD), (**C**) malondialdehyde (MDA), and (**D**) myeloperoxidase (MPO) in liver tissue. (**E**) TUNEL immunofluorescence was performed to assess hepatocyte apoptosis (original magnification: ×100 and ×200), with quantification of the relative integrated fluorescence signal. (**F**) TUNEL immunohistochemistry was conducted to evaluate hepatocyte apoptosis (original magnification: ×100 and ×200), with calculation of the percentage of positive area. Data are presented as the mean ± standard error (SEM). *p < 0.05, **p < 0.01

Analysis of network pharmacology-based studies revealed that oxidative stress exerts a pivotal role in acute liver injury and hepatocyte apoptosis triggered by Con A, which is in agreement with prior findings that have shown that Mog V has regulatory effects on multiple oxidative stress indicators [29]. Consequently, we analyzed the indicators of oxidative stress in each of the treatment groups, the findings of which revealed that Mog V pretreatment promoted significant increases in the activities of hepatic GSH and SOD (Fig. 3A, B), thereby effectively reducing the levels of Con A-induced hepatic MDA and MPO (Fig. 3C, D). To further verify the effects on liver cell apoptosis, liver tissue sections from mice were examined using TUNEL immunofluorescence and immunohistochemistry. The mice in the Mog V combined with Con A treatment group had a markedly lower number of TUNEL-positive cells in their hepatic tissues than those treated with Con A alone (Fig. 3E, F). To clarify how Mog V regulates apoptotic pathways in Con A-induced liver injury, we used Western blotting to detect core apoptosis-related proteins in liver tissue. Compared with the Control group, the Con A group showed significantly increased expression of the pro-apoptotic protein Bax and cleaved caspase-3 (an apoptosis execution marker), while the level of the anti-apoptotic protein Bcl-2 was decreased. After Mog V intervention, the levels of Bax and cleaved caspase-3 were reduced, while Bcl-2 expression was restored; no significant difference in pro-caspase-3 was observed across groups. These data indicate that Mog V exerts hepatoprotective effects by regulating the Bcl-2/Bax-caspase-3 apoptotic pathway—specifically, by enhancing anti-apoptotic signals and suppressing caspase-dependent apoptosis—thereby inhibiting hepatocyte apoptosis (Supplementary Fig. 4A, B). These findings indicate that Mog V pretreatment effectively protects liver cells, ameliorates oxidative stress levels, and inhibits liver cell apoptosis.

### Mog V Attenuates Con A-induced Liver Injury by Limiting M1 Macrophage Polarization

**Fig. 4:**
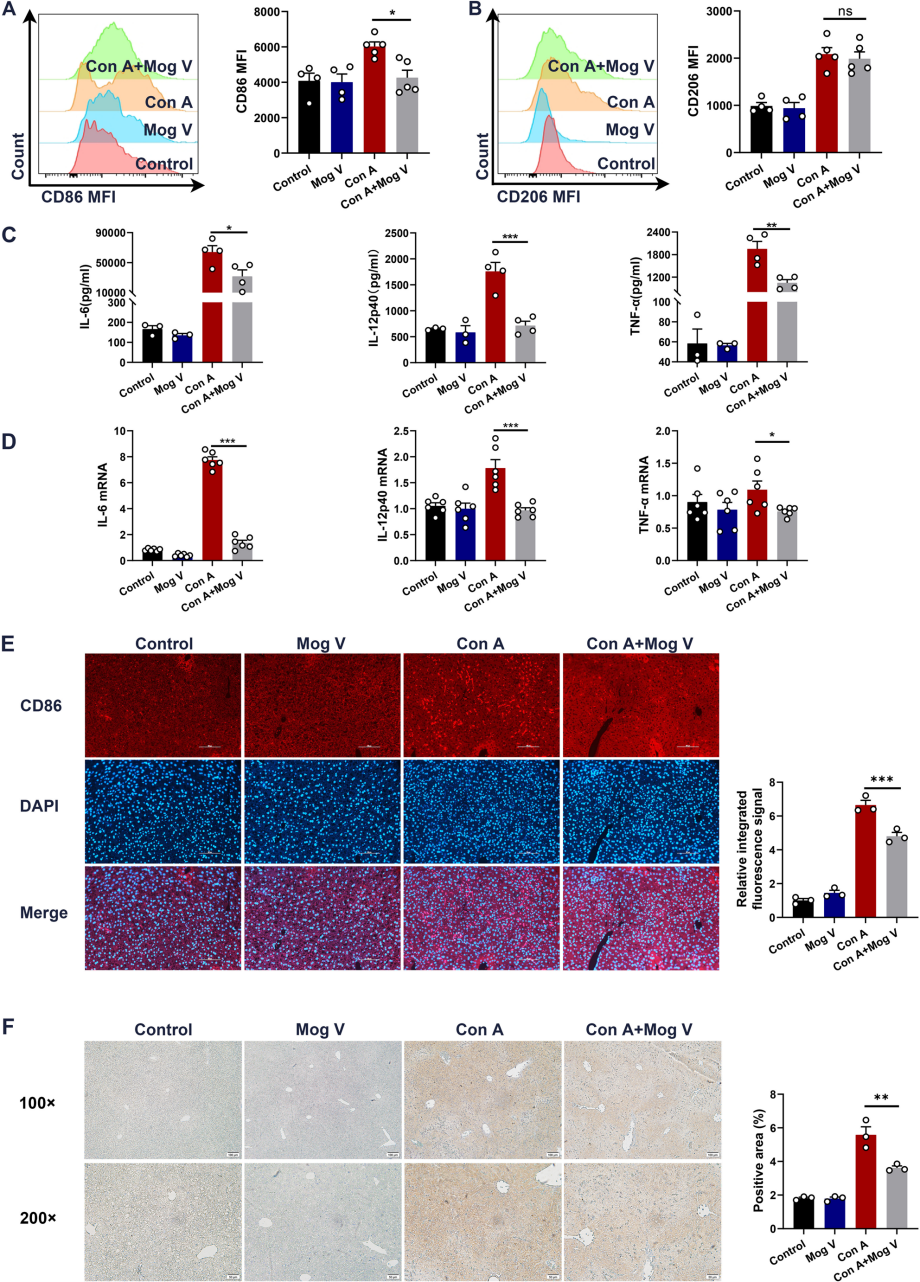
Mog V regulates M1 macrophage activation. Mice were intraperitoneally injected with Mog V (2.5, 5, or 10 mg/kg), followed by tail vein injection of Con A (20 mg/kg) 3 h later. Serum and liver tissues were collected 12 h post-Con A administration. (**A**) Mean fluorescence intensity (MFI) of CD86 (M1 macrophage marker) in hepatic macrophages. (**B**) Mean fluorescence intensity (MFI) of CD206 (M2 macrophage marker) in hepatic macrophages. (**C**) Enzyme-linked immunosorbent assays of IL-6, IL-12, and TNF-α levels in mouse serum. (**D**) qRT-PCR analysis of IL-6, IL-12, and TNF-α mRNA expression in mouse liver tissues. (**E**) Immunofluorescence staining of CD86 (red fluorescence) and DAPI (blue fluorescence) in liver tissues, along with merged images (original magnification: ×200), and quantification of the relative integrated fluorescence signal. (**F**) Immunohistochemical staining of CD86 in liver sections (original magnification: ×100 and ×200), with quantification of the positive area percentage. Data are presented as the mean ± standard error (SEM). ns: Not significant, *p < 0.05, **p < 0.01, ***p < 0.001

Given that acute hepatic injury caused by Con A is usually characterized by the activation of T and NKT cells and the release of cytokines, which induce inflammatory responses and massive hepatocyte damage [30], we further investigated the role of Mog V in immune cell activation. We found that Mog V inhibits the upregulated expression of CD69, a surface activation marker on NKT cells and T cells, induced by Con A (Supplementary Fig. 5A–C). We also assessed the effects of Mog V on myeloid-derived suppressor cells (MDSCs). The experimental data illustrated that the Mog V-treated and Con A-treated groups did not exhibit significantly different distributions of MDSCs and their subtypes (Supplementary Fig. 6A–D). The gating strategy used in this experiment is detailed in Supplementary Fig. 7A, B. In this context, we speculate that the phenotypic differentiation of macrophages may be associated with the microenvironment and that this plasticity enables macrophages to respond specifically to pathogens or certain signaling molecules [31]. Both damaged tissues and activated lymphocytes release specific signaling molecules. As key regulators of the hepatic immune microenvironment, macrophages serve as pivotal hubs linking innate and adaptive immunity, and their crosstalk with T cells and NKT cells plays a critical role in the pathogenesis of Con A-induced liver injury. Our preliminary findings demonstrated that Mog V significantly reduced activation of T cells and NKT cells in the injured liver (Supplementary Fig. 5). Given that numerous studies have confirmed that macrophages can modulate the recruitment, activation, and function of these two types of immune cells, this observation prompted us to further explore the underlying upstream regulatory mechanisms. Flow cytometric analysis was performed to quantify the expression of CD11b and CD86, markers of M1 macrophages, and CD11b and CD206, markers of M2 macrophages, across the different experimental groups. Mog V reduced the macrophage proportion in mice (Supplementary Fig. 8A). Further testing revealed that Mog V treatment significantly reduced the elevation in the mean fluorescence intensity (MFI) of M1 macrophages within the Con A-induced hepatic macrophage population (Con A + Mog V group: 4264 ± 525.8, n = 4 for control group, n = 5 for Con A‑treated group; *P* < 0.05) (Fig. 4A), while exerting no significant effect on the MFI of M2 macrophages (Con A + Mog V group: 1988 ± 171.1, n = 4 for control group, n = 5 for Con A‑treated group; *P* > 0.05) (Fig. 4B). The gating strategies are presented in Supplementary Fig. 8B–D. To further validate the hypothesis that Mog V may help protect the liver from injury induced by Con A by modulating the polarization of M1 macrophages, we examined M1 macrophage-associated pro-inflammatory factors and their gene expression levels in hepatic tissues and serum using ELISA and qRT-PCR techniques, respectively. Mog V markedly decreased the Con A-induced increase in inflammatory cytokine release (Fig. 4C, D). Immunofluorescence and immunohistochemical staining revealed that Con A promoted an increase in the percentage of M1 macrophages, which was diminished by Mog V treatment (Fig. 4E, F). These results indicate that the protective effects of Mog V in Con A-induced acute liver injury are mainly associated with the inhibition of M1 macrophage polarization and activation in mice.

### Mog V Suppresses M1 Macrophage Activity, Inflammatory Cytokine Expression and Oxidative Stress In Vitro

**Fig. 5:**
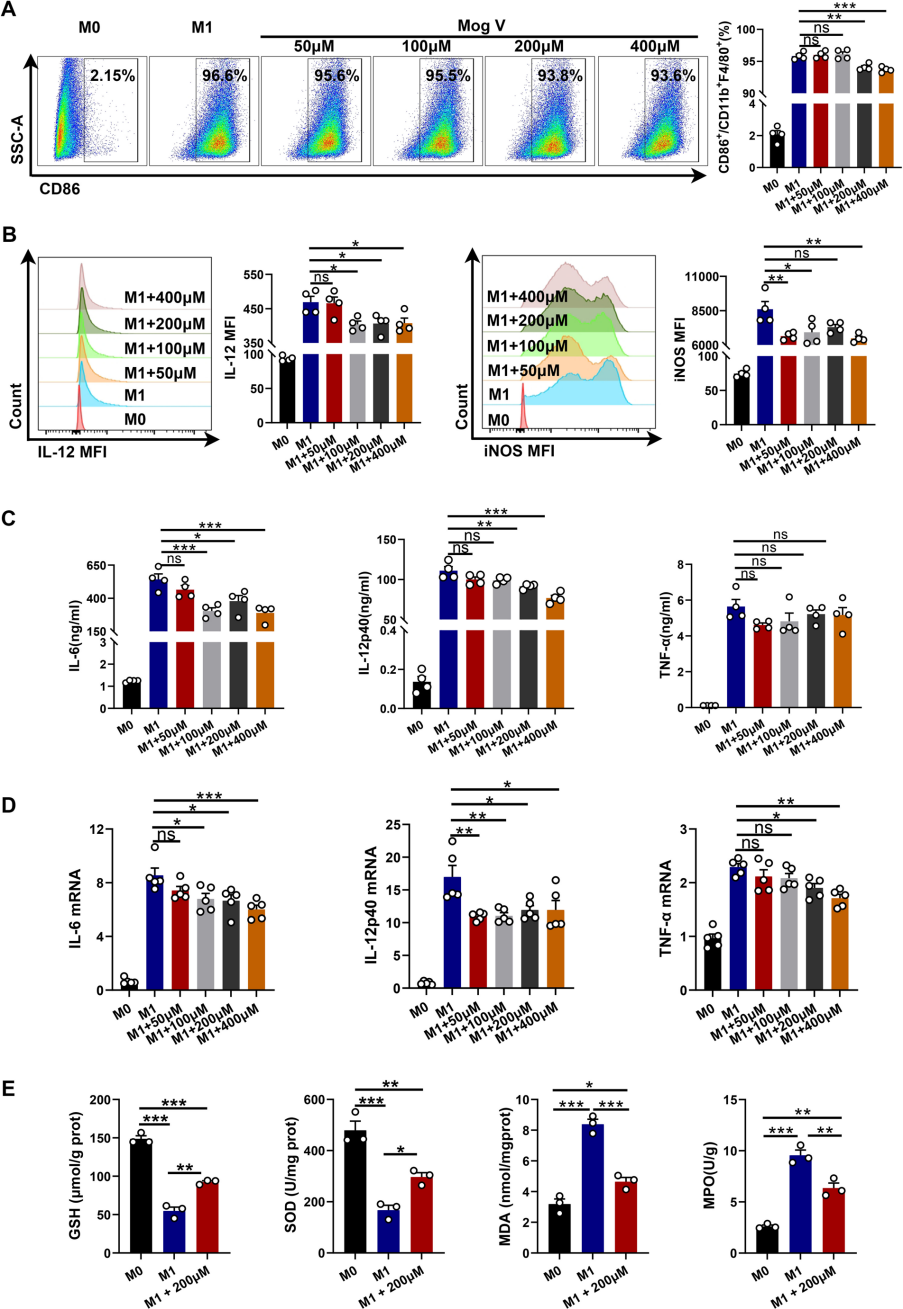
Mog V inhibits M1 macrophage activation and associated inflammatory cytokine levels in vitro. Treat bone marrow-derived macrophages (BMDMs) with Mog V; 50, 100, 200, or 400 µM) for 3 hours, then stimulate them with lipopolysaccharide (LPS) and interferon-γ (IFN-γ). Collect cell supernatants and cells after 24 hours. (**A**) Proportion of M1 macrophages (CD11b^+^F4/80^+^CD86^+^) in cultured bone marrow-derived macrophages (BMDMs) in vitro. (**B**) Mean fluorescence intensity (MFI) of IL-12 and inducible nitric oxide synthase (iNOS) in M1 macrophages in vitro. (**C**) Enzyme-linked immunosorbent assays of IL-6, IL-12, and TNF-α levels in cell culture medium. (**D**) qRT-PCR analysis of IL-6, IL-12, and TNF-α mRNA expression in cells. (**E**) Levels of GSH, SOD, MDA and MPO in macrophages. Data are presented as the mean ± standard error (SEM). ns: Not significant, *p < 0.05, **p < 0.01, ***p < 0.001

To verify our preliminary findings, we cultured macrophages under laboratory conditions and added Mog V at various concentrations (50, 100, 200, 400, 800, and 1000 µM). After 3 h, IFN-γ and LPS were used to stimulate M1 macrophages, and the effect was monitored. The toxicity of Mog V on the cells was then determined using a CellTiter-Lumi Luminescent Cell Viability Assay kit (Supplementary Fig. 9). Based on these results, we used Mog V at concentrations of 50, 100, 200, and 400 µM, with no apparent effects on cell viability. Next, we examined the proportions of CD86^+^ cells, IL-12, and iNOS, which have been identified as key factors in M1 macrophage activation, via flow cytometry. The gating strategy is shown in Supplementary Fig. 10A–C. Mog V treatment reduced the proportion of CD86^+^ cells and significantly reduced the levels of iNOS and IL-12 (Fig. 5A, B). We also examined the expression levels of M1 macrophage-related inflammatory factors, including TNF-α, IL-6, and IL-12, using ELISA and qRT-PCR and found that Mog V pretreatment contributed to reductions in the expression of these cytokines (Fig. 5C, D). Furthermore, to directly assess the regulatory effect of Mog V on oxidative stress in M1-polarized macrophages, we detected intracellular levels of MPO, MDA, SOD, and GSH. Mog V treatment significantly decreased the contents of MPO and MDA, and markedly increased the activities of SOD and GSH in activated M1 macrophages (Fig. 5E). Collectively, our findings indicate that Mog V effectively inhibited M1 macrophage activity and reduced the expression of associated inflammatory cytokines in vitro.

### Protective Effects of Mog V Treatment on Macrophage Adoptive Transfer in Liver Injury

**Fig. 6:**
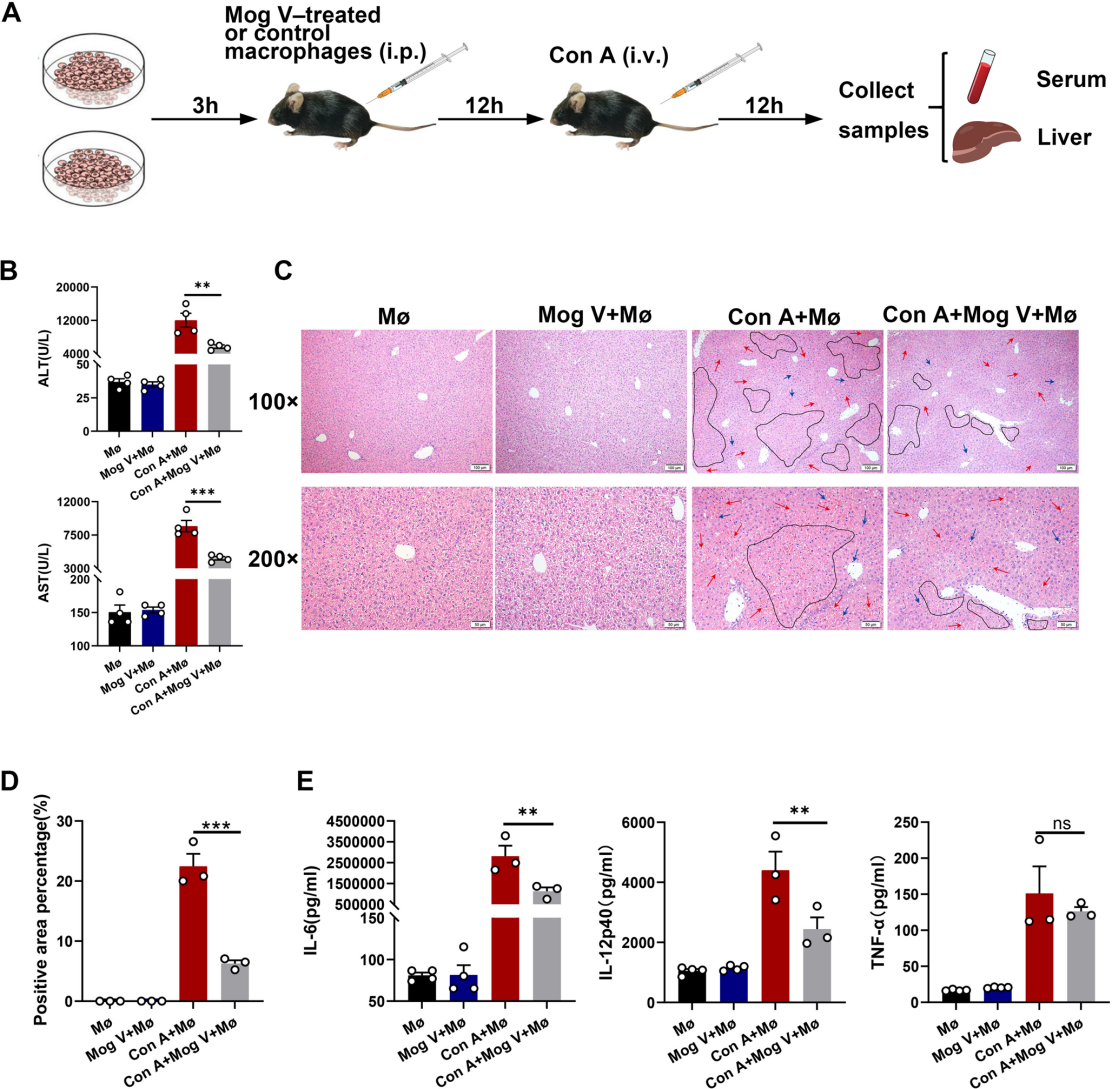
Mog V-treated macrophages attenuate Con A-induced acute liver injury. Treat macrophages cultured in vitro with Mog V. After 3 h, harvest the macrophages and intraperitoneally inject them into mice (2 × 10⁶ cells/mouse). Twelve hours later, challenge the mice with Con A (20 mg/kg) via tail vein injection. After an additional 12 h, collect serum samples and liver tissues. (**A**) Flow chart of drug administration. (**B**) Serum alanine aminotransferase (ALT) and aspartate transaminase (AST) levels. (**C**) Liver sections stained with hematoxylin and eosin (H&E), magnification: ×100 and ×200, Black dashed lines mark areas of necrosis, red arrows indicate areas of congestion, and blue arrows point to sites of inflammatory cell infiltration. (**D**) Quantitative analysis of the percentage of pathological lesion areas in H&E-stained liver sections. (**E**) Serum levels of IL-6, IL-12, and TNF-α determined using enzyme-linked immunosorbent assays. Data are presented as the mean ± standard error (SEM). ns: Not significant, **p < 0.01, ***p < 0.001

We performed in vivo adoptive transfer experiments to confirm the protective roles of Mog V-treated macrophages. A flowchart of the adoptive transfer procedure is presented in Fig. 6A. A marked reduction in serum AST and ALT levels was detected in the macrophage cell group following the addition of Mog V (Fig. 6B). H&E staining of mouse liver tissue revealed that Mog V treatment effectively protected the liver, demonstrating a marked reduction in hepatocyte necrosis (Fig. 6C, D). The protein expression levels of key cytokines (TNF-α, IL-6, and IL-12) associated with the inflammatory response in M1 macrophages were measured. Although Mog V exerted no significant regulatory effect on TNF-α expression, the protein expression levels of both IL-12 and IL-6 were reduced (Fig. 6E). Mog V-pretreated macrophages exert a protective effect against Con A-induced acute liver injury in mice by suppressing M1 macrophage polarization.

### Mog V Inhibits M1 Macrophage Polarization by Inhibiting MAPK, NF-κB, and IRF-5 Signaling Pathways

**Fig. 7:**
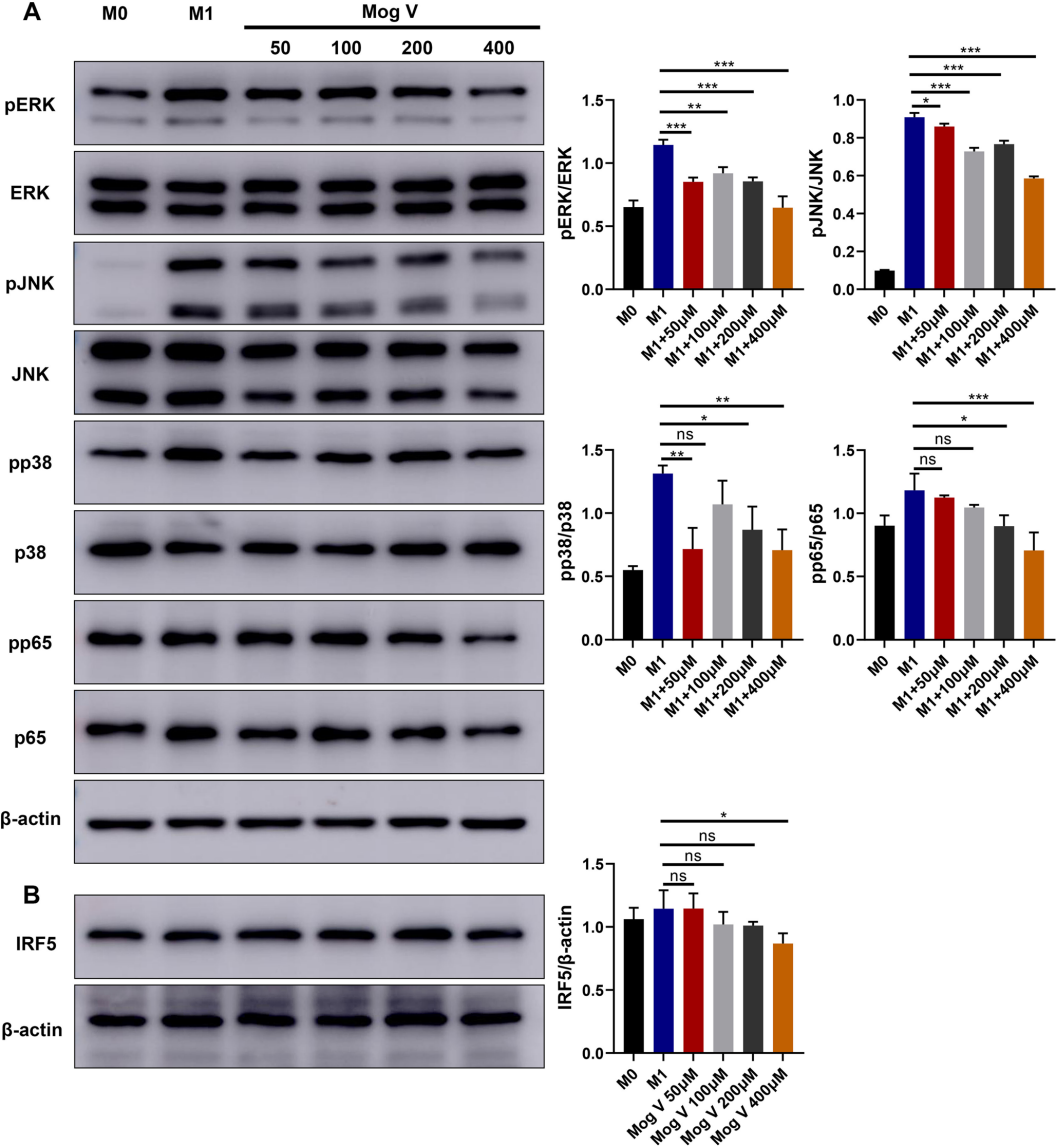
Mog V inhibits M1 macrophage activation by regulating the MAPK, NF-κB, and IRF5 signaling pathways. Bone marrow-derived macrophages (BMDMs) were treated with Mog V (50, 100, 200, or 400 µM) for 3 hours, followed by stimulation with lipopolysaccharide (LPS) and interferon-γ (IFN-γ). Cells were harvested 1h post-LPS+IFN-γ stimulation to detect molecules related to the mitogen-activated protein kinase (MAPK) and nuclear factor-κB (NF-κB) signaling pathways. Cells were harvested 24 hours post-LPS+IFN-γ stimulation to detect interferon regulatory factor 5 (IRF5). (**A**) Western blot analysis and statistics of JNK, ERK, p38, and p65 and their phosphorylation levels. (**B**) Western blot analysis and statistics of IRF5. Data are presented as the mean ± standard error (SEM). ns: Not significant, *p < 0.05, **p < 0.01, ***p < 0.001. See Supplementary Fig. 11 for complete uncropped blots

The upregulated expression of M1 macrophage-associated inflammatory elements was highly correlated with active MAPK and NF-κB signaling [32]. Moreover, the NF-κB signaling pathway was linked to Con A-induced liver injury and macrophage inflammatory processes [33, 34]. Consequently, we hypothesized that the protective effects of Mog V in Con A-induced liver injury may be mediated by the modulation of these pathways. To verify this assumption, we used western blotting to examine whether Mog V regulates macrophages through these pathways. Mog V markedly suppressed the phosphorylation of p38, ERK, NF-κB (p65), and JNK, However, we did not detect a remarkable change in the overall levels of these signaling molecules (Fig. 7A). Furthermore, considering the key role of IRF5 in the differentiation of M1-type macrophages, we further analyzed the activation level of IRF5 and detected a reduction in expression in response to Mog V treatment (Fig. 7B). Collectively, these findings indicate that Mog V inhibits M1 macrophage polarization in vitro by modulating multiple signaling pathways (NF-κB, MAPK, and IRF5), which in turn inhibits oxidative stress, apoptosis, and inflammatory reactions.

## Discussion

Acute liver injury is a progressive inflammatory disease that currently lacks effective treatment. Con A-induced liver injury models are a well-established system for studying immune-mediated acute liver injury. Previous research revealed that intravenous injection of Con A activated T cells in mice, triggering the aggregation of other immune cells, such as Kupffer cells and monocytes. These activated cells release inflammatory factors, such as IL-1, IL-6, and TNF-α, which further accelerate the progression of acute liver injury [35, 36]. This process is associated with elevated ALT and AST levels, inflammatory cell infiltration, and hepatocyte apoptosis, ultimately leading to hepatic dysfunction. Mog V, an active constituent of the plant *S. grosvenorii* (Luo Han Guo), exerts a wide range of medicinal properties, such as modulation of oxidative stress [37] and neuronal protection [38], and has potential application in diabetes therapy [39]. In this study, we examined the potential mechanisms of Con A-mediated Mog V protection in acute liver injury. The protective and therapeutic effects of Mog V against liver injury were associated with reduced levels of serum aminotransferase levels, hepatocyte apoptosis, and inflammatory cytokine expression. A notable observation in our study is that increasing Mog V concentrations beyond 5 mg/kg did not further reduce serum ALT/AST levels, indicating a plateau effect in its hepatoprotective efficacy. We hypothesize that this phenomenon may stem from the saturation of Mog V’s downstream target signaling pathways, including NF-κB, MAPK, and IRF5. As a common pharmacological characteristic, once the drug concentration reaches a threshold that fully occupies the binding sites of key target proteins in these pathways, additional increments in dosage are unlikely to enhance the therapeutic response, which aligns with our experimental findings.

Based on the network pharmacology analysis, we identified nine key genes that are both targets of Mog V and implicated in active liver injury. These nine genes included *HSP90AA1* and *IGF1*, which have been established to play roles in cellular autophagy and inflammatory processes [40]. EGFR signaling has been demonstrated to regulate survival, proliferation, and polarization in macrophages [41]. Hypoalbuminemia can be regarded as a marker of heightened systemic and local inflammation, thus serum ALB levels are closely associated with inflammatory states. The inflammatory microenvironment (high levels of TNF-α and IL-1β) is a key factor driving macrophage polarization toward the M1 phenotype. Activation of ESR1 exhibits distinct anti-inflammatory and hepatoprotective effects [42]. Our qPCR results indicate that Mog V modulates the levels of EGFR, ALB, and ESR1, thereby exerting a protective effect (Supplementary Fig. 3). A previous study revealed that IGF1 mediates DNA damage repair and decreases oxidative stress via the *IGF1R/PI3K/AKT* signaling pathway [43]. In Con A models of liver injury, this condition is associated with a decrease in antioxidant activity, including that of SOD and GSH, along with elevated levels of lipid peroxidation, as indicated by increases in MDA content [44]. These abnormalities in oxidative stress indicators lead to mitochondrial DNA damage, thereby triggering hepatocyte apoptosis and promoting the expression of inflammatory factors. Previous research has shown that Mog V modulates riboflavin (vitamin B2) metabolism and may thus influence its role in regulating oxidative stress [19, 45]. In line with the KEGG pathway (Fig. 2C) and GO enrichment (Fig. 2D) analyses from our network pharmacology study, Mog V is predicted to primarily target the oxidative stress metabolic pathway, with the NF-κB signaling axis serving as its core mediating node—consistent with the significant enrichment of the “response to reactive oxygen species” process, a key metabolic event. While direct pathway intervention experiments were not performed in this study, existing literature supports that blocking this pathway (e.g., via antioxidants) alleviates acute liver injury, which mirrors Mog V’s protective phenotype, whereas activating it (e.g., via exogenous reactive oxygen species) exacerbates damage, which opposes Mog V’s effects [46]. These findings collectively suggest the oxidative stress metabolic pathway is a key target of Mog V; future in vitro/in vivo experiments (e.g., pathway inhibitor/activator intervention, target protein manipulation) will validate the causal link between this pathway and Mog V-induced phenotypes. Our findings indicate that the protective effects of Mog V in Con A-induced acute liver injury are closely linked to the regulation of hepatic oxidative stress and related metabolic pathways in mice. MDA accumulation causes cellular damage, and its levels in cells are a crucial indicator of the degree of oxidative stress [47]. The use of MPO inhibitors (heme-containing peroxidase) can reduce inflammatory responses [48]. SOD is a core antioxidant enzyme secreted by hepatocytes and Kupffer cells [49], whereas GSH is a tripeptide that maintains intracellular redox homeostasis [50]. In this study, we measured the levels of oxidative stress indicators in the liver following Mog V treatment and examined hepatocyte apoptosis in mouse liver tissue sections using the TUNEL method. Pretreatment with Mog V led to marked reductions in the levels of liver MPO and MDA induced by Con A and enhanced the activities of GSH and SOD, indicating that Mog V alleviates oxidative damage and protects hepatocytes from apoptosis.

To confirm that this regulation of oxidative stress directly occurs in macrophages rather than merely reflecting secondary effects from hepatocytes, we further measured oxidative stress indicators in M1-polarized macrophages in vitro. We found that Mog V directly attenuated oxidative stress in macrophages, as evidenced by decreased MPO and MDA levels and increased SOD and GSH activities. These data verified that macrophages are important target cells of Mog V in regulating oxidative stress during acute liver injury. Macrophages, particularly those derived from monocytes and Kupffer cells, are key players in the inflammatory responses associated with liver injury [51], where they function as highly diversified phagocytes that play indispensable immune roles in the body [52]. Macrophages are not only pleiotropic, with the capacity to undergo transition from one phenotype to another [53], but they are also highly adaptive, responding to different microenvironmental stimuli in diverse tissue types [54]. In acute liver injury, macrophages are typically polarized to the pro-inflammatory M1 type, which activates hepatic stellate cells and releases large quantities of pro-inflammatory factors, including cyclooxygenase-2, iNOS, and TNF-α, thereby exacerbating hepatitis progression [55]. Hepatic macrophages initiate adaptive immune responses by releasing pro-inflammatory cytokines such as tumor necrosis factor-α (TNF-α) and interleukin-1β (IL-1β), which activate dendritic cells (DCs), upregulate the expression of co-stimulatory molecules on DCs, and induce DCs to secrete chemokines including C-C motif chemokine ligand 5 (CCL5) and C-C motif chemokine ligand 19 (CCL19) [56, 57], thereby mediating the recruitment of circulating T cells to liver tissues [58]. In addition, interleukin-6 (IL-6) and interleukin-12 (IL-12) secreted by hepatic macrophages can promote the differentiation of naive T cells into pro-inflammatory T helper 17 (Th17) and T helper 1 (Th1) subsets, respectively, thus amplifying the hepatic inflammatory cascade [59, 60]. Conversely, interleukin-10 (IL-10) secreted by regulatory T cells (Tregs) can feedback-inhibit M1 polarization of macrophages and reduce the levels of IL-6 and TNF-α secreted by macrophages—this cellular crosstalk maintains immune homeostasis during the resolution of liver inflammation and tissue repair [61]. For NKT cells, a key subset of innate-like T cells, macrophages function as antigen-presenting cells by expressing CD1d molecules, which present self-lipid antigens to activate NKT cells [62]. Activated NKT cells rapidly secrete large amounts of cytokines such as interferon-γ (IFN-γ), which in turn enhance the pro-inflammatory activity of macrophages, forming a pathogenic positive feedback loop. Collectively, these studies confirm that macrophages are the core upstream regulators governing the recruitment and activation of T cells and NKT cells in the Con A-induced liver injury model. Based on our experimental observation that Mog V reduces the proportions of T cells and NKT cells in the liver, we propose the following scientific hypothesis: Mog V may exert its hepatoprotective effects by targeting macrophages—its mechanism of action may be either directly regulating macrophage polarization and function, or indirectly modulating the responses of T cells and NKT cells by disrupting the crosstalk between macrophages and these immune cells. To verify this hypothesis, we subsequently focused our research on investigating the regulatory role of Mog V in macrophage polarization and its downstream effects on hepatic inflammation. Our findings revealed that Mog V treatment was associated with M1 and M2 macrophage activation within the liver, as confirmed by flow cytometry. In both serum and liver tissue sections, we detected a marked decrease in the expression of inflammatory cytokines associated with M1 macrophages (e.g., TNF-α and IL-6). Mog V pretreatment reduces the mRNA expression levels of IL-6 and TNF-α in liver tissues, while the protein levels of these cytokines detected by ELISA in liver homogenates show a relatively mild reduction; this phenomenon may be related to post-transcriptional/translational regulation in macrophages, or the compensatory secretion of pro-inflammatory cytokines by other immune cells (e.g., T cells and NKT cells) that are partially regulated by Mog V. Beyond regulating macrophage polarization, Mog V suppresses Con A-induced upregulation of CD69 on T cells and NKT cells, supporting its coordinated regulation of both cell populations. By inhibiting T/NKT cell activation and subsequent IFN-γ secretion (which amplifies macrophage pro-inflammatory activity), this intercellular crosstalk mediates Mog V’s anti-inflammatory phenotype, clarifying that its protective effects extend beyond macrophages through multi-immune cell interactions. These observations provide evidence that Mog V reduces M1-type macrophage activation and may shift macrophage polarization toward an M2 type, which is associated with tissue damage and inflammation reduction. To further investigate the effects of Mog V on macrophage polarization, M1-type macrophages were cultured in vitro and treated with different concentrations of Mog V. In agreement with our in vivo findings, flow cytometry indicated that Mog V treatment markedly reduced the percentage of M1 macrophages in vitro. Notably, we observed that intraperitoneally (*i.p*.) injected macrophages accumulated more in the livers of control mice than in Con A-induced liver injury mice, despite the lack of an inflammatory microenvironment in the control group. This phenomenon can be attributed to three key factors: (1) physiological hepatic sequestration, where the intact liver structure in homeostatic conditions efficiently traps *i.p*.-injected macrophages; (2) inflammatory disruption of liver architecture in Con A-induced injury, which impairs the liver’s ability to retain macrophages; and (3) macrophage redistribution, where the inflammatory milieu redirects *i.p*.-injected macrophages to other inflamed tissues or accelerates their clearance. These observations provide additional context for interpreting the macrophage adoptive transfer results and highlight the dynamic nature of macrophage trafficking in the context of liver injury. Our findings from the macrophage adoptive transfer assay indicated that Mog V protects the liver by suppressing M1 macrophage polarization and decreasing pro-inflammatory cytokine discharge.

The activation of macrophages that characterize acute inflammatory diseases is generally associated with the modulation of key signaling pathways, such as IRF, NF-κB, and MAPK [33]. In this regard, Mog V has been reported to alleviate LPS-mediated neuroinflammatory responses by preventing NF-κB and MAPK signaling pathways in BV-2 cells, thereby highlighting the anti-inflammatory capacity of Mog V [38]. However, to the best of our knowledge, no prior research has investigated the effect of Mog V on liver injury-related signaling pathways. The target of action of Mog V and its association with genes involved in the development of acute liver injury were investigated based on network pharmacological analyses. The MAPK and NF-κB pathways were identified as key signaling pathways. Mog V treatment markedly reduced the phosphorylation of key signaling molecules, including CASP3, EGFR, and IGF1, as well as the transcription factor IRF5, which is essential for macrophage infiltration and motility [63]. Subsequent western blot analysis confirmed that Mog V suppresses the phosphorylation of proteins in the IRF5, MAPK, and NF-κB signaling pathways, thereby contributing to reduced inflammation and hepatocyte death, preventing acute liver injury triggered by Con A.

Collectively, our findings demonstrate that Mog V exerts preventive and therapeutic protective effects against Con A-induced acute liver injury in mice by synergistically alleviating hepatic oxidative stress, inhibiting M1 macrophage polarization, and suppressing the activation of NF-κB, MAPK and IRF5 inflammatory signaling pathways. These findings provide experimental evidence for the potential application of Mog V in the treatment of immune-mediated acute liver injury, and further in-depth studies (e.g., large animal models, clinical trials) are required to evaluate its clinical translation potential.

Although this study investigated both the protective and therapeutic effects of Mog V, it has certain limitations. First, the research was conducted solely using the C57BL/6J mouse model of concanavalin A-induced acute liver injury; the protective effects and underlying mechanisms of Mog V have not been verified in other animal models or clinical samples. Additionally, the in vivo adoptive transfer experiment of macrophages only assessed short-term effects at 24 h, and the long-term protective efficacy as well as its impact on mouse survival rate remain to be further explored. Second, the study focused exclusively on the regulatory effect of Mog V on M1 macrophage polarization, without investigating its potential impacts on other immune cells such as dendritic cells and regulatory T cells, nor the crosstalk among these relevant cells. Furthermore, while several key targets including ALB, EGFR and ESR1 were predicted via network pharmacology, the direct binding relationships and specific regulatory mechanisms between these targets and Mog V have not yet been validated.

Future studies will focus on verifying the protective effects of Mog V in other animal models and clinical samples, exploring its regulatory effects on multiple immune cells and cell-cell crosstalk, and confirming the direct binding and regulatory mechanisms between Mog V and the predicted key targets through molecular docking, co-immunoprecipitation and other experimental techniques.

## Conclusions and Future Directions

**Fig. 8:**
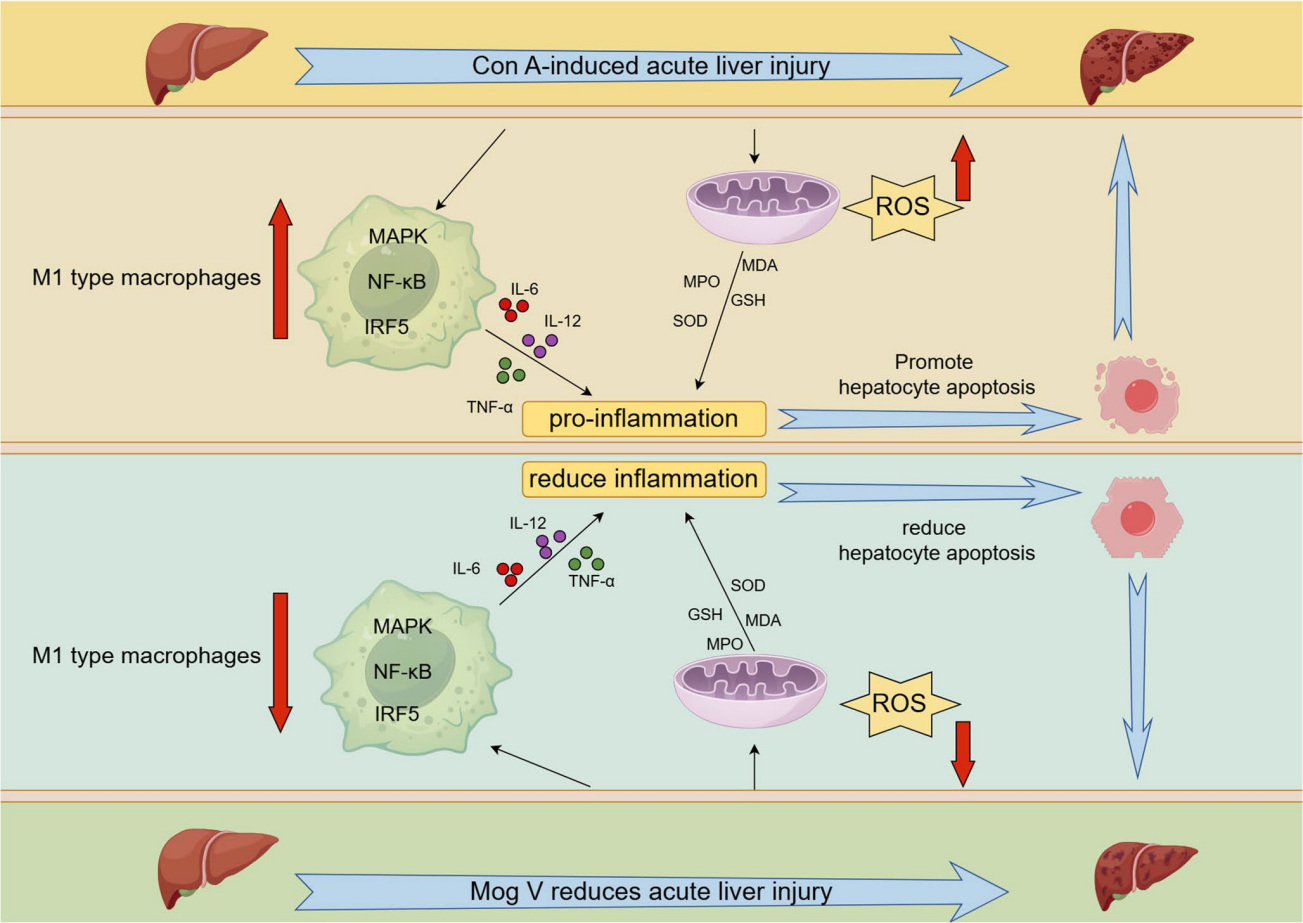
Potential mechanisms of Mog V action on Con A-induced acute liver injury. Mog V protects mice from Con A-induced acute liver injury by inhibiting M1 macrophage activation and reducing the levels of associated inflammatory factors via modulation of the MAPK, NF-κB, and IRF5 signaling pathways, and attenuating inflammation by regulating oxidative stress

This study demonstrates that Mog V, an active component of *Siraitia grosvenorii*, exerts both protective and therapeutic effects against Con A-induced acute liver injury in mice. We found that Mog V reduced serum transaminase levels, alleviated hepatocyte apoptosis, and decreased the expression of pro-inflammatory cytokines. Its mechanisms may be related to the inhibition of IRF5, NF-κB, and MAPK signaling pathway activation, thereby suppressing M1 macrophage polarization and activation, as well as regulating oxidative stress (Fig. 8). Meanwhile, Mog V enhanced antioxidant enzyme activities and reduced the levels of oxidative stress markers MDA and MPO. These multiple effects collectively mediate the hepatoprotective effects of this compound (Fig. 8). These findings provide experimental evidence for the potential of Mog V as a novel candidate for the development of therapeutic agents against immune-mediated acute liver injury. Future studies will focus on clarifying the direct molecular targets of Mog V, verifying its protective effects in more animal models, and exploring its pharmacokinetic characteristics and safety, so as to lay a solid foundation for its subsequent clinical research and application. Future studies should focus on clarifying the molecular mechanisms of Mog V and assessing its clinical application.

## Supplementary Information

Below is the link to the electronic supplementary material.

Supplementary Material 1 (DOCX 6.30 MB)

## Data Availability

The data are available from the corresponding author upon reasonable request.
