# Supplementary material for: Mogroside V Alleviates Concanavalin A-induced Acute Liver Injury by Inhibiting Inflammatory Responses and M1 Macrophage Polarization
Source: Inflammation. 2026 Mar 29;49(1):136. doi: 10.1007/s10753-026-02493-8 (PMC13172022; doi:10.1007/s10753-026-02493-8)
Supplement: Supplementary file 1 — Supplementary Material 1 (DOCX 6.30 MB) [file 10753_2026_2493_MOESM1_ESM.docx]

**Mogroside V alleviates concanavalin A-induced acute liver injury by inhibiting inflammatory responses and M1 macrophage polarization**

**Yuxuan Zhao^a,1^, Zhihong Liu^a,b,1^, Fenglian Yan^a,c^, Hongru Zhao^a^, Xinzhou Xie^d^, Jiaying Li^a^, Hui Zhang^a,c^, Lin Wang^a^, Jia Fu^e^, Chunxia Li^a,c^, Jun Dai^a,c^, Huabao Xiong^a,^*, Bin Yu ^f,^*and Junfeng Zhang^a,^***

^a^Institute of Immunology and Molecular Medicine, Jining Medical University, Jining 272067, China

^b^School of Basic Medicine, Shandong First Medical University, Jinan 271016, China

^c^Jining Key Laboratory of Immunology, Jining Medical University, Jining 272067, China

^d^Department of Spine Surgery, Jining First People's Hospital, Jining 272011, China

^e^Basic Medical College, Jining Medical University, Jining 272067, China

^f^College of Integrated Chinese and Western Medicine, Jining Medical University, Jining 272067, China

^1^Yuxuan Zhao and Zhihong Liu contributed equally to this work.

***Corresponding authors:**

Junfeng Zhang, E-mail: zjfart001@163.com; Tel.: +86 0537-3616283

Bin Yu, E-mail: yubin@mail.jnmc.edu.cn; Tel.: +86 0537-3616158

Huabao Xiong, E-mail: xionghbl@163.com; Tel.: +86 0537-3616283

**Supplementary Figures**

**
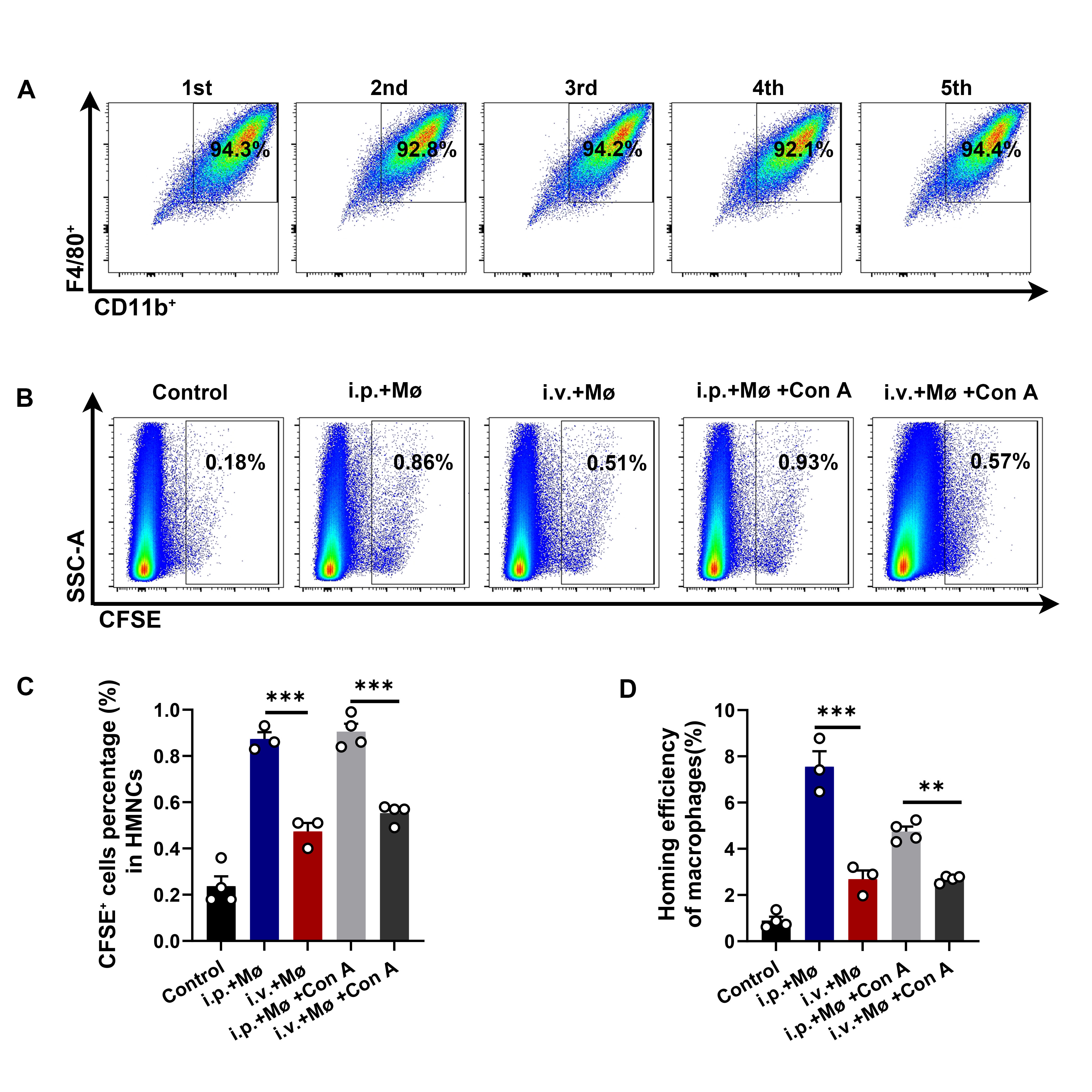
**

**Supplementary Fig. 1. Intraperitoneal injection of macrophages exhibited a higher hepatic homing efficiency than tail vein injection.** Label 1×10⁶ cells/mL BMDMs with 1 μM CFSE in the dark for 20 min. Inject 2×10⁶ labeled macrophages per mouse intraperitoneally (i.p.) or intravenously (i.v.). Twelve hours later, induce liver injury by intravenous injection of Con A. Euthanize mice an additional 12h later and isolate liver tissue. Analyze the proportion of CFSE⁺ cells in liver single-cell suspensions using flow cytometry. Homing efficiency (%) = (Number of CFSE⁺cells in liver / Total number of injected CFSE⁺ macrophages) × 100%. A. Flow cytometry results from five independent replicate experiments demonstrate that the purity of induced macrophages remains consistently above 90%. B. CFSE⁺cells percentage (%) in HMNCs. C. Statistical results of CFSE⁺ cells percentage (%) in HMNCs. D. Statistical results on the homing efficiency of adoptively transferred macrophages, homing efficiency (%) = (Number of CFSE⁺ cells in liver / Total number of injected CFSE⁺ macrophages) × 100%. Data are presented as the mean ± Standard Error of the Mean (SEM). ***p < 0.001.

**
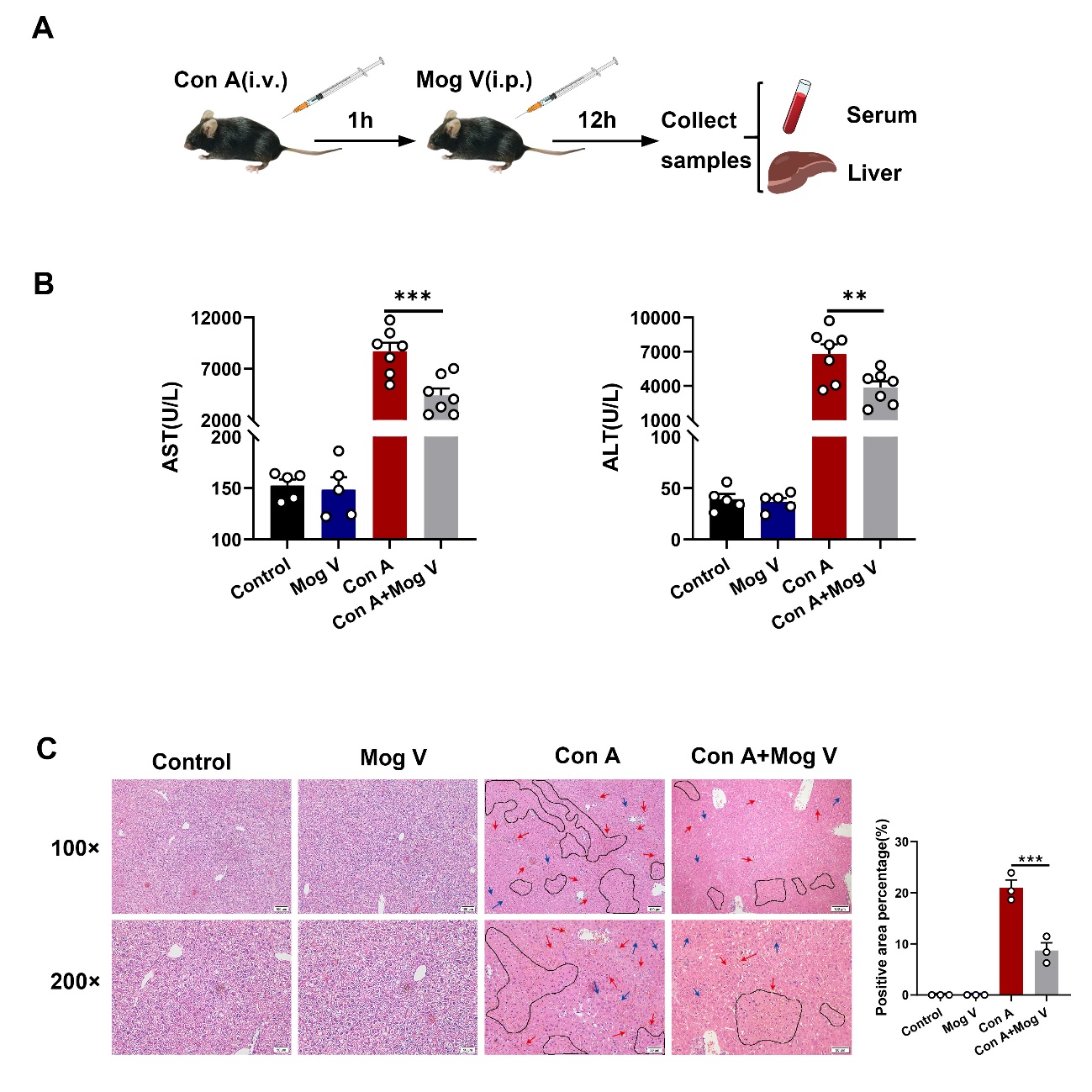
**

**Supplementary Fig. 2. Mog V retains its hepatoprotective effects when administered after the establishment of the injury model.** One hour after tail vein injection of Con A (20 mg/kg) in mice, administer Mog V (5 mg/kg) via intraperitoneal injection. Collect serum and liver tissue 12 hours later. A. Flow chart of drug administration. B. Serum aspartate transaminase (AST) levels and Serum alanine aminotransferase (ALT) levels. C. Quantitative analysis of the percentage of pathological lesion areas in H&E-stained sections (original magnification: ×100 and ×200) and quantitative analysis percentages of H&E-positive areas, black dashed lines mark areas of necrosis, red arrows indicate areas of congestion, and blue arrows point to sites of inflammatory cell infiltration. Data are presented as the mean ± SEM. **p < 0.01, ***p < 0.001.


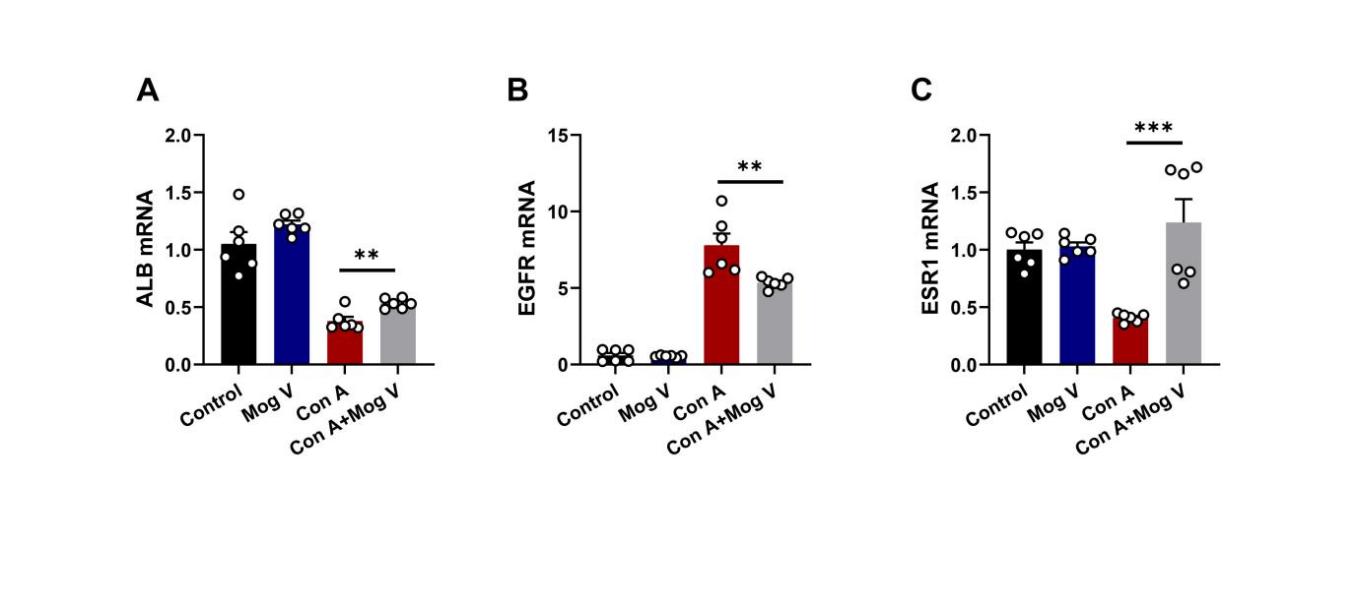


**Supplementary Fig. 3. qRT-PCR analysis of mRNA expression levels for key target genes ALB, EGFR, and ESR1 in network pharmacology screening.** A. mRNA levels of ALB. B. mRNA levels of EGFR. C. mRNA levels of ESR1. Data are presented as the mean ± SEM. **p < 0.01, ***p < 0.001.


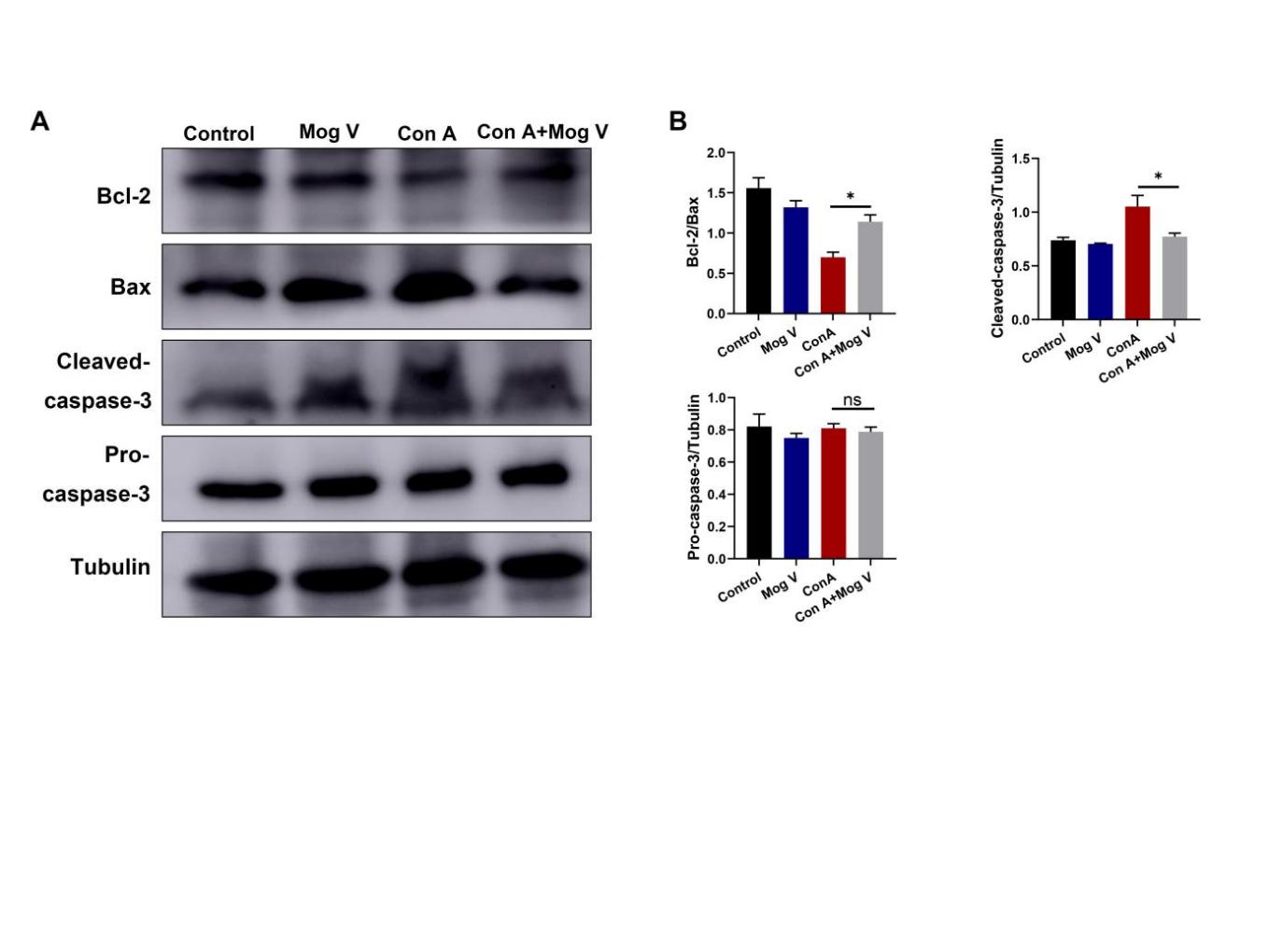


**Supplementary Fig. 4. Mog V Inhibits Con A-Induced Hepatocyte Apoptosis by Regulating Apoptosis-Related Protein Expression.** Mice received intraperitoneal injections of Mog V followed by Con A challenge 3 hours later. Liver tissue was collected 12 hours post-challenge to detect the expression of proteins associated with hepatocyte apoptosis. A. Western blot analysis of Bcl-2, Bax, Cleaved-caspase-3, Pro-caspase-3, and Tubulin in mouse liver tissue. B. Statistical analysis of apoptosis-related protein expression levels (including Bcl-2/Bax ratio, Cleaved-caspase-3, and Pro-caspase-3) in mouse livers across groups. Data are presented as the mean ± SEM. ns: Not significant, *p < 0.05.


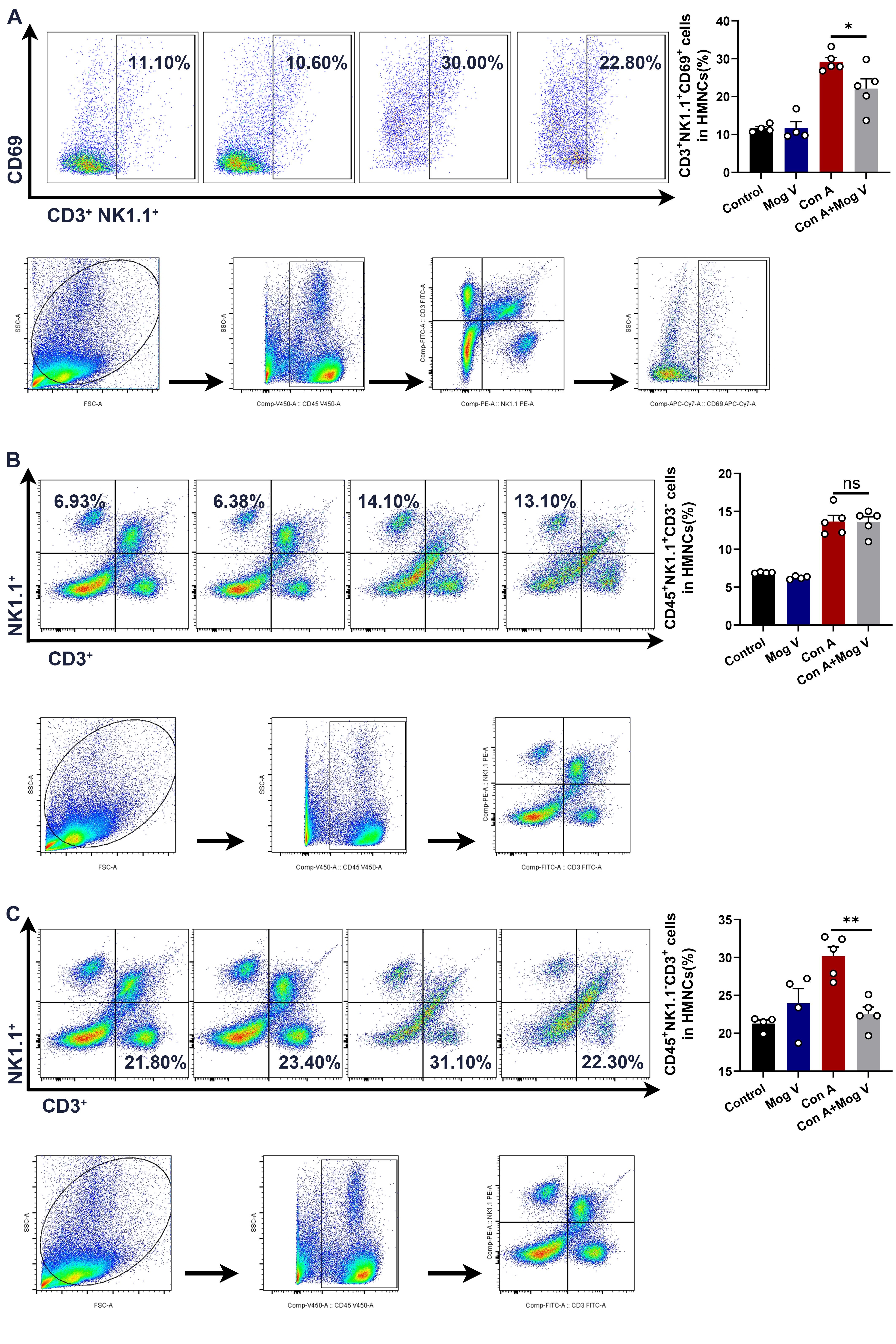


**Supplementary Fig. 5.** **Effects of Mog V on hepatic natural killer T (NKT) cells, natural killer (NK) cells, and T cell activation**. A. Activation of NKT cells (CD3^+^NK1.1^+^CD45^+^) and gating strategy. B. Activated NK cells (CD45⁺ NK1.1⁺ CD3⁻) and gating strategy. C. Activated T cells (CD45⁺ NK1.1⁻ CD3⁺) and gating strategy. Data are presented as the means ± SEM. ns: Not significant, *p < 0.05, **p < 0.01.


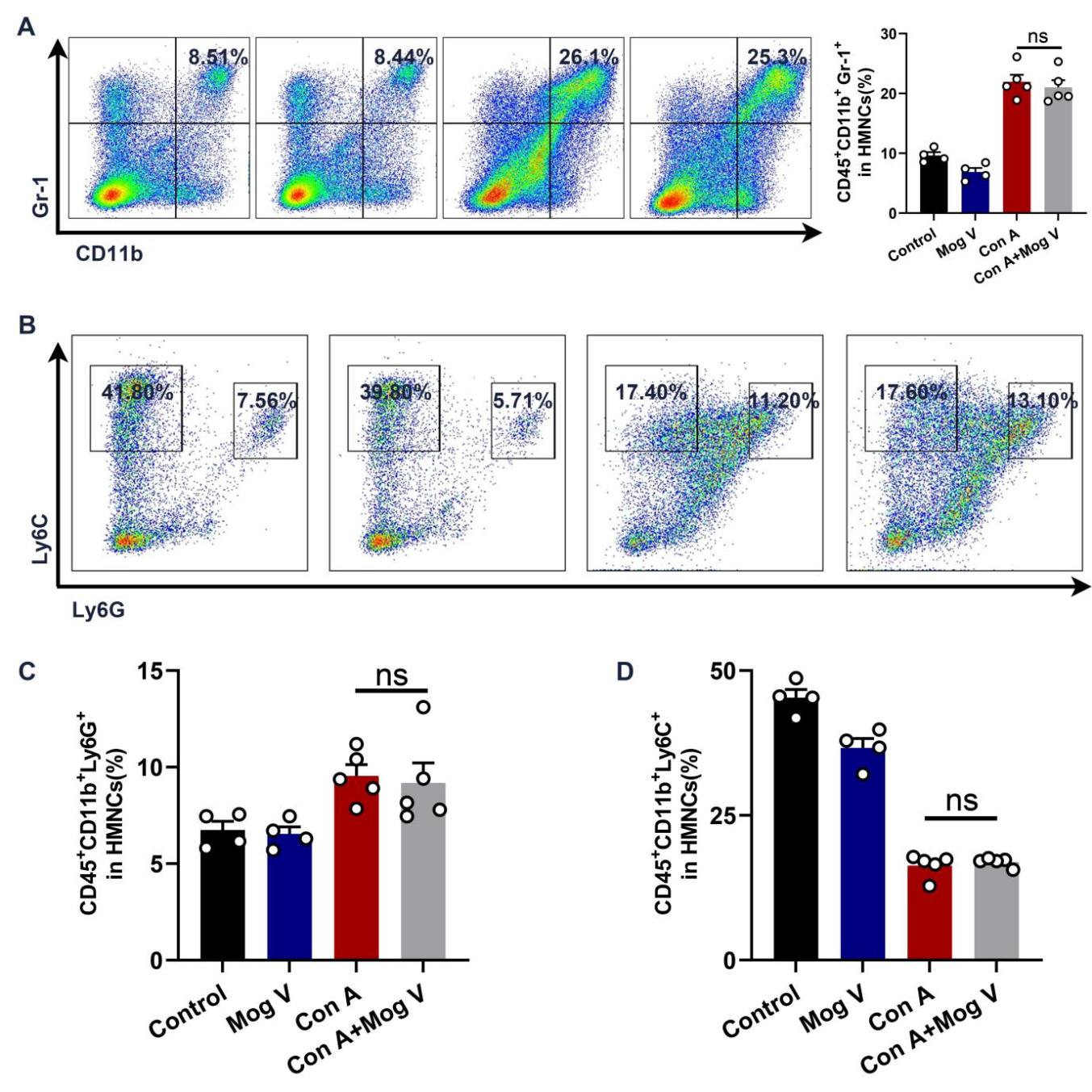


**Supplementary Fig. 6.** **Effects of** **Mog V on hepatic myeloid-derived suppressor cells (MDSCs)**. A. Percentage of MDSCs (CD45^+^CD11b^+^Gr-1^+^) in hepatic mononuclear cells (HMNCs) and its statistical results. B. Percentages of G-MDSCs (Ly6G^+^Ly6C^－^) and M-MDSCs (Ly6G^－^Ly6C^+^). C. Statistical results of G-MDSC ratios. D. Statistical results of M-MDSC ratios. Data are presented as the means ± SEM. ns: Not significant.


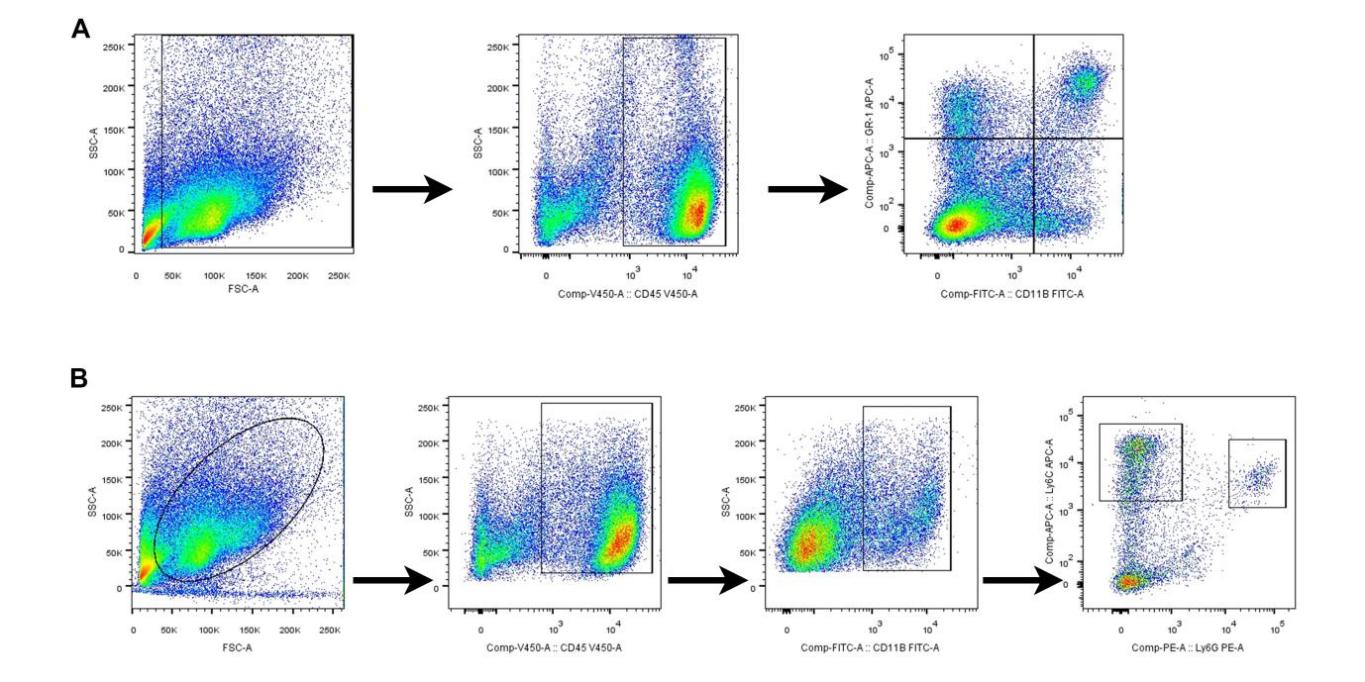


**Supplementary Fig. 7.** A. Detailed gating strategy for Supplementary Fig. 6A. B. Detailed gating strategy for Supplementary Fig. 6B.


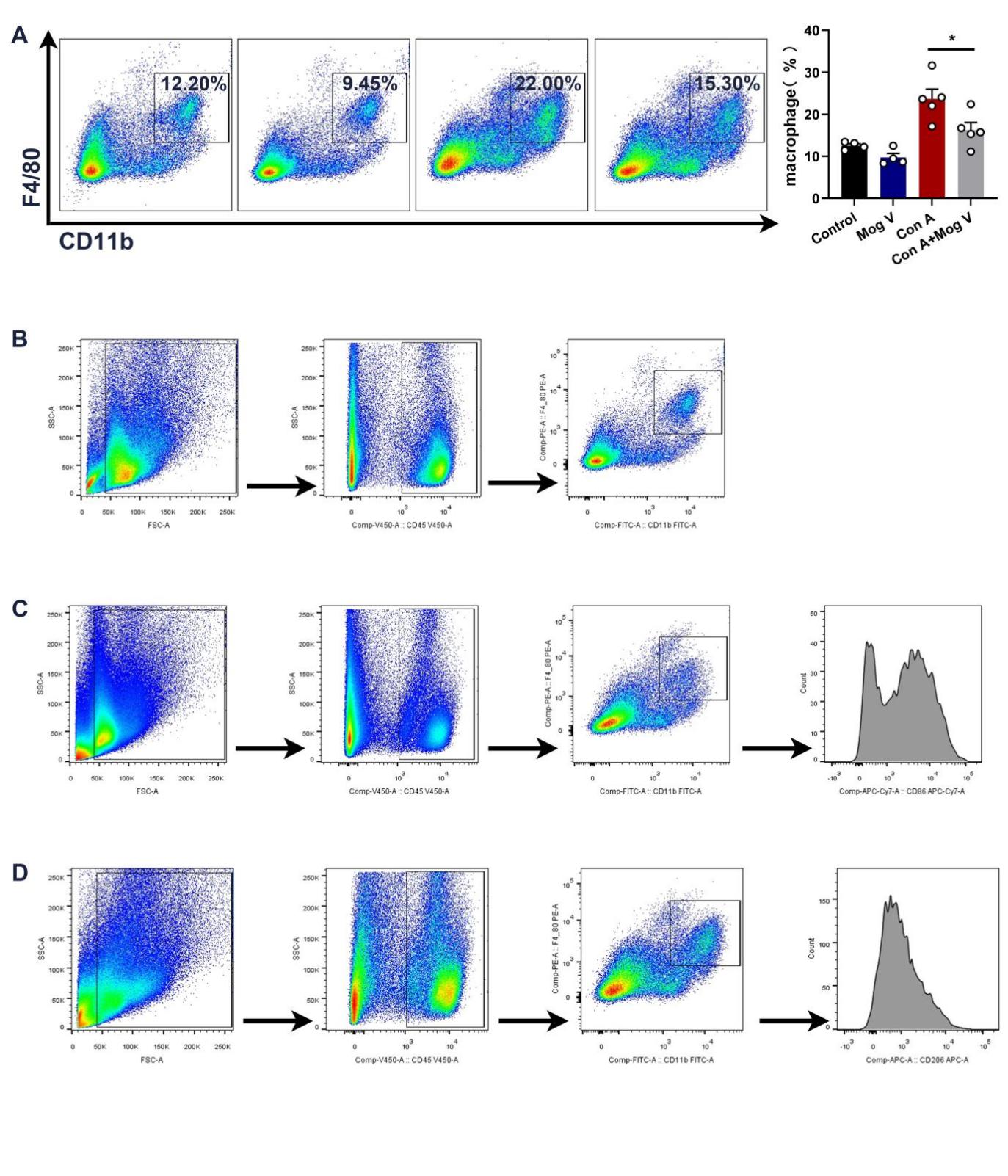


**Supplementary Fig. 8.** A. Percentage of hepatic macrophages (CD11b^+^F4/80^+^) in HMNCs and its statistical results. Data are presented as the means ± SEM, *p < 0.05. B. Detailed gating strategies for Supplementary Fig. 8A. C. Detailed gating strategies for Fig. 4A D. Detailed gating strategies for Fig. 4B.


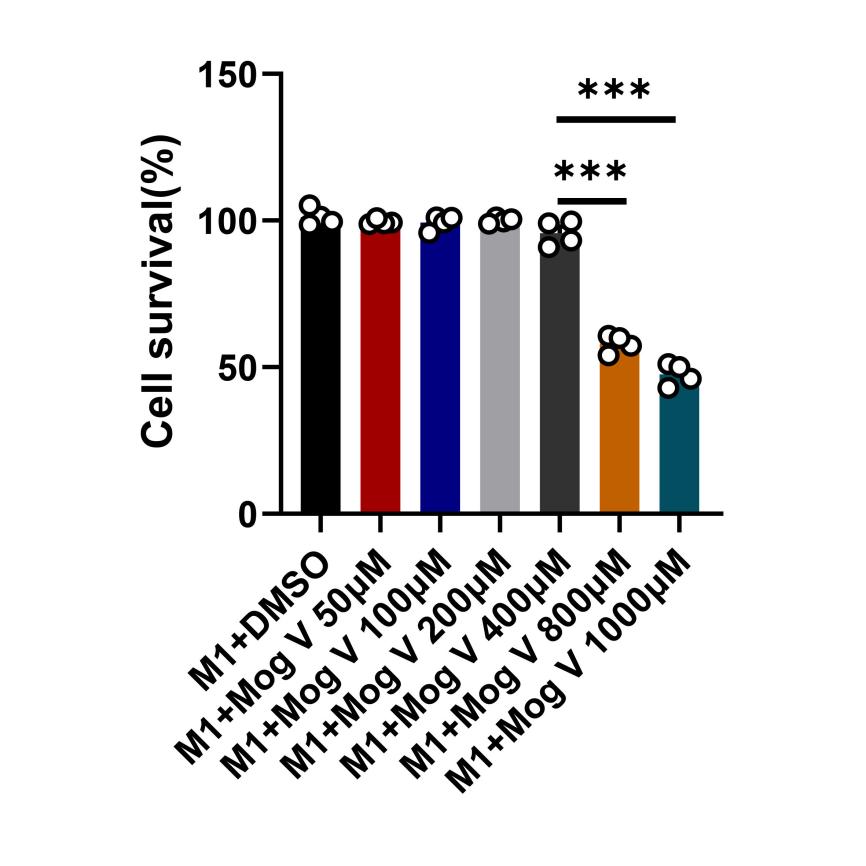


**Supplementary Fig. 9.** Toxic effects of different concentrations of Mog V (50, 100, 200, 400, 800, and 1000 µM) (with M1+DMSO as the control group) on bone marrow-derived macrophages detected using a Cell Counting Kit-8 assay. Data are presented as the means ± SEM. ***p < 0.001.


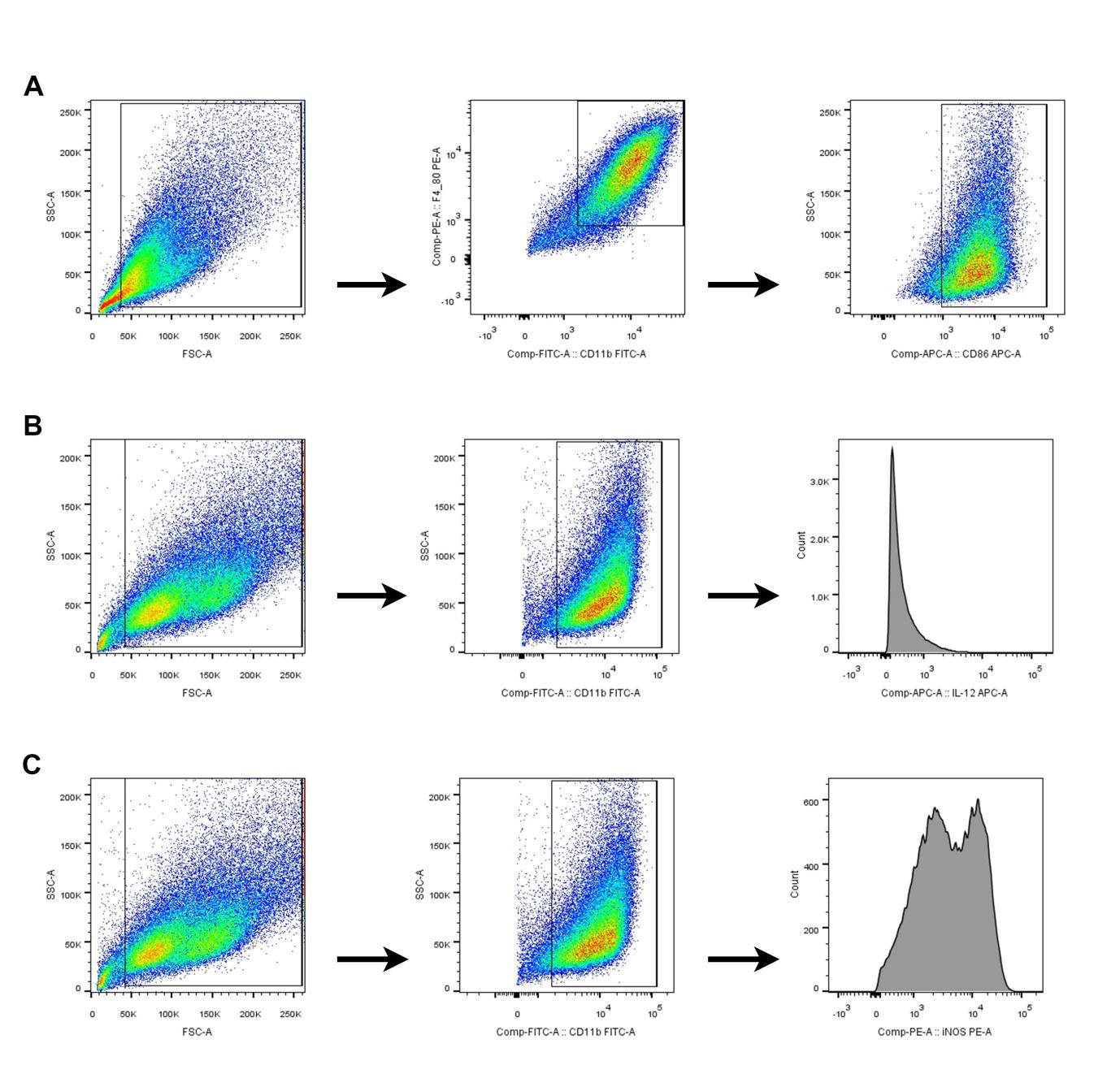


**Supplementary Fig. 10.** A. Detailed gating strategies for the macrophage population in Fig. 5A. B. Gating process (left panels) and IL-12 mean fluorescence intensity (IL-12 MFI; right panel) corresponding to Fig. 5B. C. Gating process (left panels) and iNOS mean fluorescence intensity (iNOS MFI; right panel) corresponding to Fig. 5B.
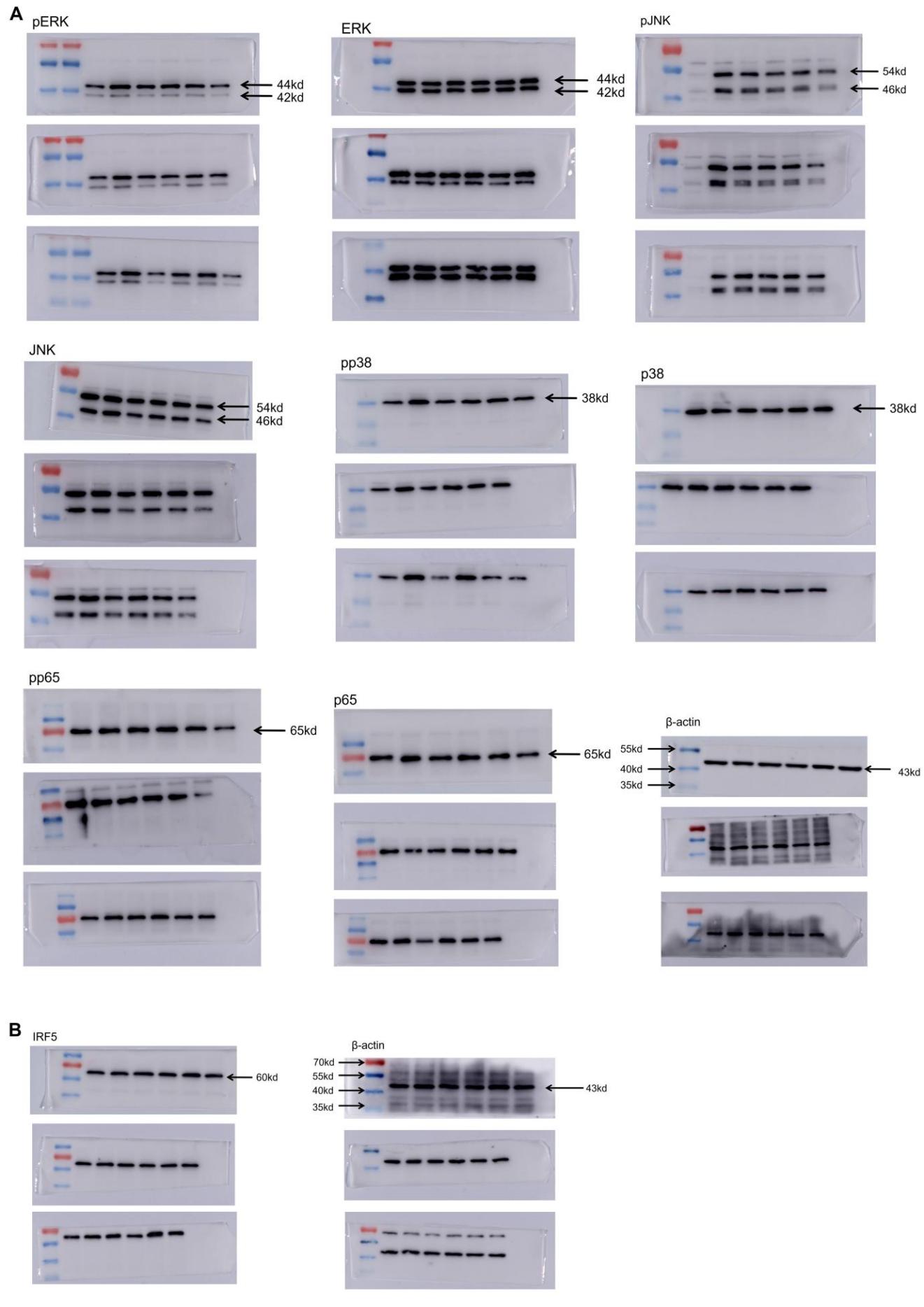


**Supplementary Fig. 11.** A. Complete, uncropped Western blot images (for pERK, ERK, pJNK, JNK, pp38, p38, pp65, p65, and β-actin) corresponding to Fig. 7A. B. Complete, uncropped Western blot images (for IRF5 and β-actin) corresponding to Fig. 7B.
